# Boats on the rocks: Late prehistoric nautical iconography and landscape, from Northwest Iberia to Scandinavia

**DOI:** 10.1371/journal.pone.0349417

**Published:** 2026-06-09

**Authors:** Marta Díaz-Guardamino, Boel Bengtsson, Ellie Newton, Ana M. S. Bettencourt, Johan Ling, Juan Latorre-Ruiz, Hugo A. Sampaio, Diogo Marinho

**Affiliations:** 1 Department of Archaeology, Durham University, Durham, United Kingdom; 2 Department of Historical Studies / Swedish Rock Art Research Archives, University of Gothenburg, Gothenburg, Sweden; 3 Department of History, Lancaster University, Lancaster, United Kingdom; 4 Department of History / Laboratory of Landscapes, Heritage and Territory (Lab2PT), Minho University, Braga, Portugal; Universidad de Sevilla, SPAIN

## Abstract

This paper offers a comparative analysis of prehistoric rock art boat depictions found across northwest Iberia and southern Scandinavia. We apply advanced digital documentation techniques, including high-resolution 3D recording and Geographic Information Systems (GIS)-based landscape analysis, to study the Iberian petroglyphs’ iconography and geographic placement in detail. The study identifies significant typological and iconographic parallels between Northwest Iberia and Nordic images, which provides a comparative basis to propose a Late Bronze Age chronology (c. 1300–800 BCE) for the Iberian examples. This shared iconography supports existing hypotheses concerning extensive long-distance connectivity and maritime trade networks across Atlantic Europe, particularly regarding the movement of metals like copper and tin. Furthermore, the GIS analysis confirms that nearly all Iberian sites, whether coastal or far inland, maintain a crucial visual or physical relationship with navigable waters, such as the ocean or major river systems. Ultimately, the authors propose that this rock art reflects both advancements in boat technology and the ritual and cosmological beliefs of maritime communities engaged in transregional exchange.

## Introduction

Bronze Age boats depicted on rocks are commonly associated with Scandinavia, where they are the most prevalent motif in rock art, with over 20,000 depictions discovered to date [1]. Recently discovered rock art sites along the Atlantic coast of Iberia, particularly in northern Portugal and southwest Galicia, feature clear or potential boat depictions that show striking similarities to Bronze Age Scandinavian boat depictions on rocks and bronze objects [2–4]. This lends credence to the hypothesis of long-distance metal exchange during the Bronze Age between Iberia, the Atlantic communities and Scandinavia [5–7]. Driven by these recent findings, Johan Ling and Marta Díaz-Guardamino, from the RAW project (*Rock Art, Atlantic Europe, Words, and Warriors*), funded by the Swedish Research Council (Vetenskapsrådet, Ref. 2018−01387), together with researchers from Portugal and Galicia (Ana M.S. Bettencourt, Hugo A. Sampaio, Diogo Marinho, Beatriz Comendador Rey), organized a field campaign in the autumn of 2021 with the following objectives: Firstly, to create cutting-edge 3D documentation of these recent finds in Portugal and Galicia using laser scanning, photogrammetry, and Reflectance Transformation Imaging (RTI) (see below); Secondly, to compare the results of this project with documentation of boats from Bronze Age Scandinavian rock art and bronze items; Finally, to see if we could find more details that could advance, falsify, or modify the previously highlighted similarities and dating. This paper presents new observations from the new digital recording of six rock art sites featuring definite or likely boat depictions: Santo Adrião, Eira do Louvado, Laje da Churra, and Senhora de Encarnação 1 in northern Portugal, as well as Penedo do Muro 1–2 and Laxe Auga dos Cebros 1 in southwest Galicia. These datasets, together with existing documentation (including recently produced records such as Escada 2 and Borna) are used to conduct a new assessment of boat imagery in northwestern Iberian rock art. Our study includes a comparative analysis of Iberian boat imagery with Scandinavian examples and synthesizes recent Geographic Information Systems (GIS)-based spatial analysis results, highlighting the relationship between these rock art boat imagery and broader patterns of connectivity.

### Rock art and connectivity in Northwest Iberia

**Fig 1:**
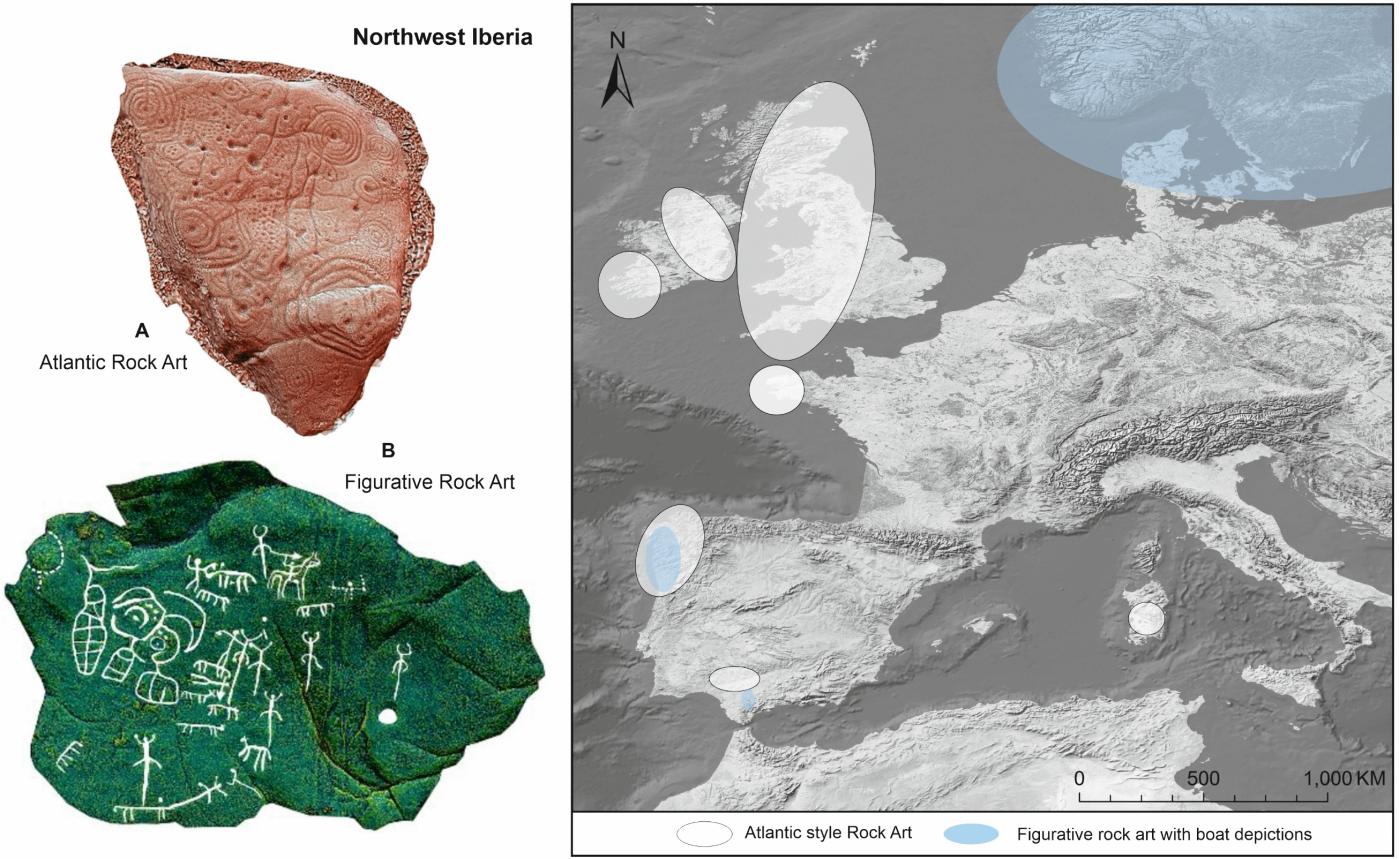
Left: Examples of Atlantic and Figurative Rock Art traditions in northwest Iberia: (A) Tapada do Ozão, Valença; and (B) Monte de Porreiras 6, Paredes de Coura, Portugal. Reprinted from [22] under a CC BY 4.0 license, with permission from Luís Coutinho (original copyright 2024). **Right: Distribution of Atlantic-style Rock Art and Figurative Rock Art with boat depictions in Europe.** Reprinted under a CC BY 4.0 license, with permission from Marta Díaz‑Guardamino, using Natural Earth raster map data (CC BY 4.0).

Thousands of prehistoric and protohistoric rock art sites with varying geometric and figurative carved and painted motifs populate the landscapes of northwest Iberia [8–21]. Traditionally, two distinctive rock art traditions have been identified in the region, Atlantic Rock Art (ARA) and Schematic Rock Art (Fig 1). In Iberia, ARA is mostly restricted to the northwest, and tends to be found on flat or low slope surfaces of granite and generally cover the whole panel or a large part of it; this rock art is part of a broader phenomenon that extends across several Atlantic regions of Europe [13,15,23–26], including southwest Iberia [27], although exceptionally. The second is Schematic Rock Art, which in northwest Iberia mainly appears in its southern and eastern margins, but is found well beyond this region, across the Iberian Peninsula, along the mountainous systems framing the Iberian Central Meseta and in the Mediterranean basin [19,24,28–31]. Schematic rock art comprises highly stylized motifs, which are mostly painted and at times engraved, mainly in the most western areas of the northwest. In painting, this style focuses strongly on scenes with schematic human figures and animals (e.g., deer, goats), and the depiction of ‘idols’, oculi and solar signs. The engravings also include several abstract motifs, such as segmented squares and U-shaped motifs, the latter dating after the Early Bronze Age [14–15]. Schematic Rock Art can be found in diverse contexts, including outcrops, rock shelters and caves, as well as megalithic monuments. Chronologically, the tradition expands from the Neolithic to the Bronze Age.

ARA is exclusively engraved and, for those who advocate a long diachrony, spans the Neolithic, Bronze Age, and the Iron Age (c. 3500–700 BCE) [9,10,13,25,28]. For these authors, there are several phases. The oldest being the Neolithic (5^th^/4^th^ and 3^rd^ millennia BCE), focusing on geometric designs, mainly cup marks, concentric circles and spirals (these can also, rarely, appear in the most western megalithic art [9–10]). During the Bronze and Iron Ages (2^nd^ millennium and beginning of the 1^st^ millennium BCE), ARA would be focused on weapons, idols, horses, deer and warriors. For other authors this is a new artistic phenomenon (Figurative Rock Art) associated with structural transformations occurring at the beginning of the Bronze Age [14,16–18,20,21,22]. In any case, it has been argued that the oldest phase of ARA offers one of the clearest indications of connectivity between northwest Iberia, Britain and Ireland, during later prehistory [23,24,26].

Although similar types of petroglyphs made with comparable techniques and placed on analogous landscape settings are found in these three regions, similar motifs of ARA can also be found in western France [32], and occasionally in southwestern Iberia [27], around the Alps [33] and Sardinia [34–35]. These distant cases could be linked to the trade of jadeite axes from the western Italian Alps—home to the well-known quarries of alpine jadeite exploited between 4700–3700 BCE—and the spread of worldviews that promoted the construction of monumental stone structures honouring both the dead and the living, as proposed by Bettencourt [18]. Indeed, links between Iberia and Brittany, where clear instances of ARA have not been yet recorded, are attested since the 5^th^ millennium BCE through the circulation of variscite and Alpine jadeite [36–39]. However, connections between Iberia, Britain and Ireland are judged to have first unfolded during the 4^th^ and 3^rd^ millennia BCE, as parallelisms between megalithic architectures and art from Ireland, northern Wales and western Iberia, especially from the late 4^th^ millennium BCE, most clearly suggest [40–42].

Connectivity between Ireland and southwestern Iberia during the 3^rd^ millennium BCE is suggested by the distribution of certain metallic artefacts [43] and indicated by recent results of Lead Isotope Analysis (LIA) of Chalcolithic artefacts from Ireland (dated to 2500–2150 BCE), which point to southwest Iberia as an initial source of copper [44]. This connectivity between Iberia and the Irish Sea could have unfolded via Brittany, primarily through down-the-line trade networks that saw the movement of materials, ideas (incl. innovations, like copper smelting) and styles, such as the Bell Beaker pottery tradition, rather than large groups of people [43,45–49]. Occasionally, some artefacts moved as imports, such as the Palmela points thought to have originated in the lower Tagus region in Iberia, which are found in Brittany and beyond [50], or the golden basket hair ornaments of Iberian style found in Britain [46,51].

During the late 3rd and early 2nd millennium BCE, a new type of rock art imagery emerges in northwest Iberia, coinciding with broader changes in settlement patterns, material culture, and funerary practices [17]. Based on this, Bettencourt [14–18] argues that this art represents not a later phase of Atlantic Rock Art (ARA), but an entirely new artistic tradition, which she terms ‘Figurative Rock Art.’ This art is mainly focused on the depiction of weapons such as daggers and halberds [16,52,53], rare flat axes, segmented circles, boats [14–18,21], and the first scenes of horses in their natural habitat [22]. This was a period of increased connectivity between northwest Iberia, Brittany and Ireland [17,44,54–56]. While LIA and elemental analysis results of some Irish Early Bronze Age (EBA) artefacts (2150–1600 BCE) are consistent with ores from southwest Iberia [44], categories and styles of metallic artefacts (e.g., halberds and daggers) manufactured locally with different compositions were shared among these three regions. Furthermore, there is something interesting about Chalcolithic and Bronze Age mining sites in Ireland and Great Orme in Wales. Strawberry tree, which is genetically linked to Iberia, is found around those mines [57]. Whether this is the result of Bronze Age human mobility is an open –but highly suggestive—question.

Connectivity between Iberia and other Atlantic regions faded during the Middle Bronze Age [56], only to be intensified during the Late Bronze Age (LBA) (c. 1300–850 BCE), particularly between northwest and western Iberia, western France, southern England, and the north Atlantic, as part of a renewed economic cycle with long-distance exchange and advanced seafaring skills [43,58–64]. During the LBA, copper and silver from southern Iberia (i.e., the mining regions of Linares, Los Pedroches and Alcudia valley) reached northwest Iberia (e.g., the silver set of Antas de Ulla in Galicia [65], copper ingots in northern Portugal, bronze palstaves in Galicia [66–67], southern England (e.g., Salcombe bay ingots [68]), and Scandinavia [4,6]. In this context, northwest Iberia became a key player in the mediation of Atlantic exchange networks. It worked as a connectivity hub/exit point for the goods (e.g., copper, silver and possibly tin) that were reaching this area from southern Iberia through a set of important terrestrial routes around the western periphery of the central Meseta, outlined by the distribution of iconography and material culture, such as the warrior stelae or bronze axes [4,69].

Nautical iconography is relatively scarce in Iberian rock art, making its concentration in two key regions particularly noteworthy [14,15,18,70–86]. These regions, northwest Iberia (the focus of this paper) and southwest Iberia, served as crucial connectivity hubs, facilitating long-distance maritime connections between the Atlantic and the Mediterranean during later prehistory. Various authors place Iberia as an important maritime junction between Atlantic and Mediterranean networks [4,60,63,87–90], which is reinforced through the geomorphological features of the coastline. Part of the boat depictions on rock art and portable artefacts could be seen as responses from local communities seeking to understand foreign travellers in an era of ‘globalization’ [5,83,91]. There are interesting parallels between the coastline of the Rías Baixas in the northwest of Iberia and the coastline of northern Britain and Scandinavia [12]. It is generally acknowledged that rock art sites with boat imagery are found in strategic locations away from domestic sites and closely related to the sea [79], although a systematic GIS-based spatial analysis of their landscape setting has not been conducted yet.

## Methods

### Rock art dating: comparative typology

Boat iconography from northwest Iberia has been mostly discussed from stylistic and chronological points of view. This has enabled establishing parallelisms between this imagery (currently known at ten sites) and Scandinavian boat depictions and therefore, linking these Iberian sites, albeit timidly, to Bronze Age Atlantic connectivity, maritime trade and knowledge transfer [14,18,62,77,79,85,86]. There are, however, various important open questions regarding the typology of the vessels depicted, associated imagery, their temporality and context within their respective panels, that can be addressed through comparison with Scandinavian boat iconography.

**Fig 2:**
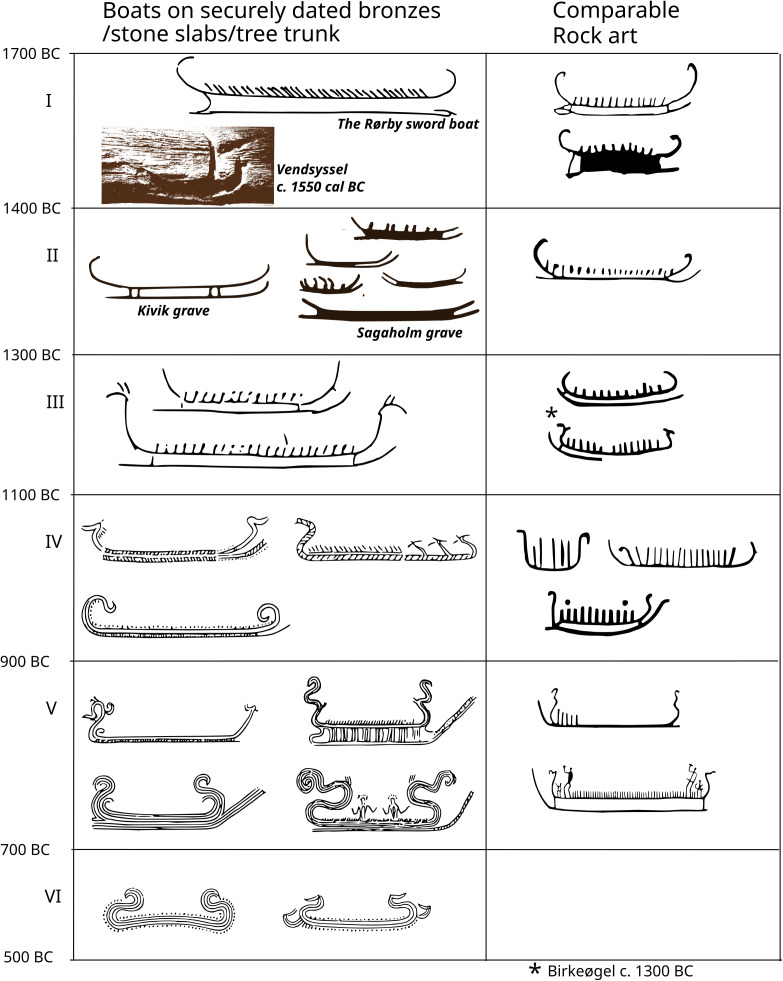
Scandinavian boat chronology adapted after Flemming Kaul [92] based on securely dated imagery in relation to examples of otherwise undatable boats within the rock art featuring the same type of end ships [93]. Rock art boats are outlined sometimes by a single line, others by a double line, and sometimes the area between the double lines are completely hollowed out. In the equivalent imagery on datable bronzes, the hull lines are often made up of multiple lines. Reprinted under a CC BY 4.0 license, with permission from Boel Bengtsson.

The southern Scandinavian boat iconography is dated in relation to chronologies built upon boat imagery appearing on bronzes and on slabs of stone found within closed grave contexts. The perhaps most famous of these include the Rørby sword from Denmark, securely dated to c. 1600 BCE, and the rock art within the Kivik and the Sagaholm graves in Sweden, securely dated to c.1400 BCE and 1400–1300 BCE, respectively [92]. The latest and now generally accepted dating chronology uses the shape of the two end ships of the depicted vessels as the main dating criteria and suggests that the main rock carving period spans the entire Bronze Age (c. 1700–500 BCE) (Fig 2) [92]. However, at some individual rock art sites the tradition seems to have persisted into the Pre-Roman Iron Age [94,95].

The general outline of the boats depicted within this imagery and the boatbuilding tradition they convey, can be traced back in time from the c. 350 BCE Hjortspring boat to the very beginning of the Scandinavian Bronze Age [96–97]. Further to this, the Scandinavian chronology seems to agree with studies of when a particular type of rock art boat could have been carved at the lowest lying rock art panels in relation to studies of local relative sea levels in the region [98–99]. In southern Norway, studies of boat carvings on several rock art panels and the succession of this boat imagery (in which order they have been carved) provide additional dating criteria for this region [95]. However, due to the inherent difficulties in connecting rock art in general to any datable context, it is necessary to always refer to the securely dated imagery when establishing a tentative date. This securely dated imagery also includes a boat image carved onto a once living tree trunk found at Vendsyssel in Denmark, which is dated to c. 1550 cal. BCE [96,100–102].

**Fig 3:**
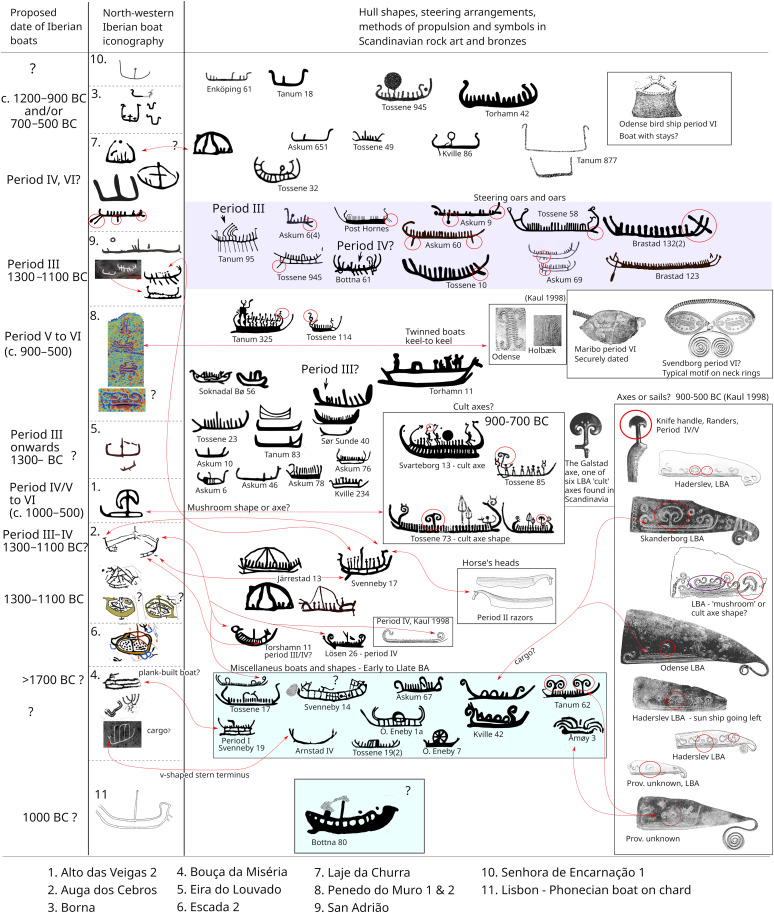
Mind map comparing western Iberian imagery (numbered 1 to 10 in alphabetical order) with Scandinavian boat imagery and symbols. The mind map is used to get an overview of the material and any potential parallels, regardless of whether the imagery can be clearly dated or not. Highlighted in red circles are mushroom/cult axe shapes and steering oars, with red arrows pointing out further parallels of interest. The potential date of the Iberian imagery in comparison to the Scandinavian material is presented in the left-hand column [84,92,95,93,103–109]. Reprinted under a CC BY 4.0 license, with permission from Boel Bengtsson.

Whereas several of the carved boat images identified at coastal and riverine locations on the Atlantic coast of the Iberian Peninsula are very rudimentary and therefore difficult to interpret, there is no doubt that there are parallels with boats that can be dated within the Scandinavian chronologies (Fig 3). In addition to end ship decorations (such as birds and s-shapes) other parallels include the inclusion of ‘mushroom’/’cult-axe’/’sail’ -like shapes located either at the centre of the boat (several parallels, with near identical such shapes located in boats at a similar location) or found decorating the end ships or pommels on bronze knives.

The perhaps most interesting parallels are offered by imagery of what is most likely larger vessels featuring what appears to be rigging for the purpose of carrying a sail (Fig 3), which not only date to similar periods but also share locations overlooking potentially important seafaring routes [93,110]. Another interesting depiction that might be useful in the discussion is a carved boat from Bottna 80, on the Swedish west coast, which appears to depict a large vessel with a similar end ship to what is interpreted as a Phoenician boat depicted on a piece of pottery from Lisbon [84].

**Fig 4:**
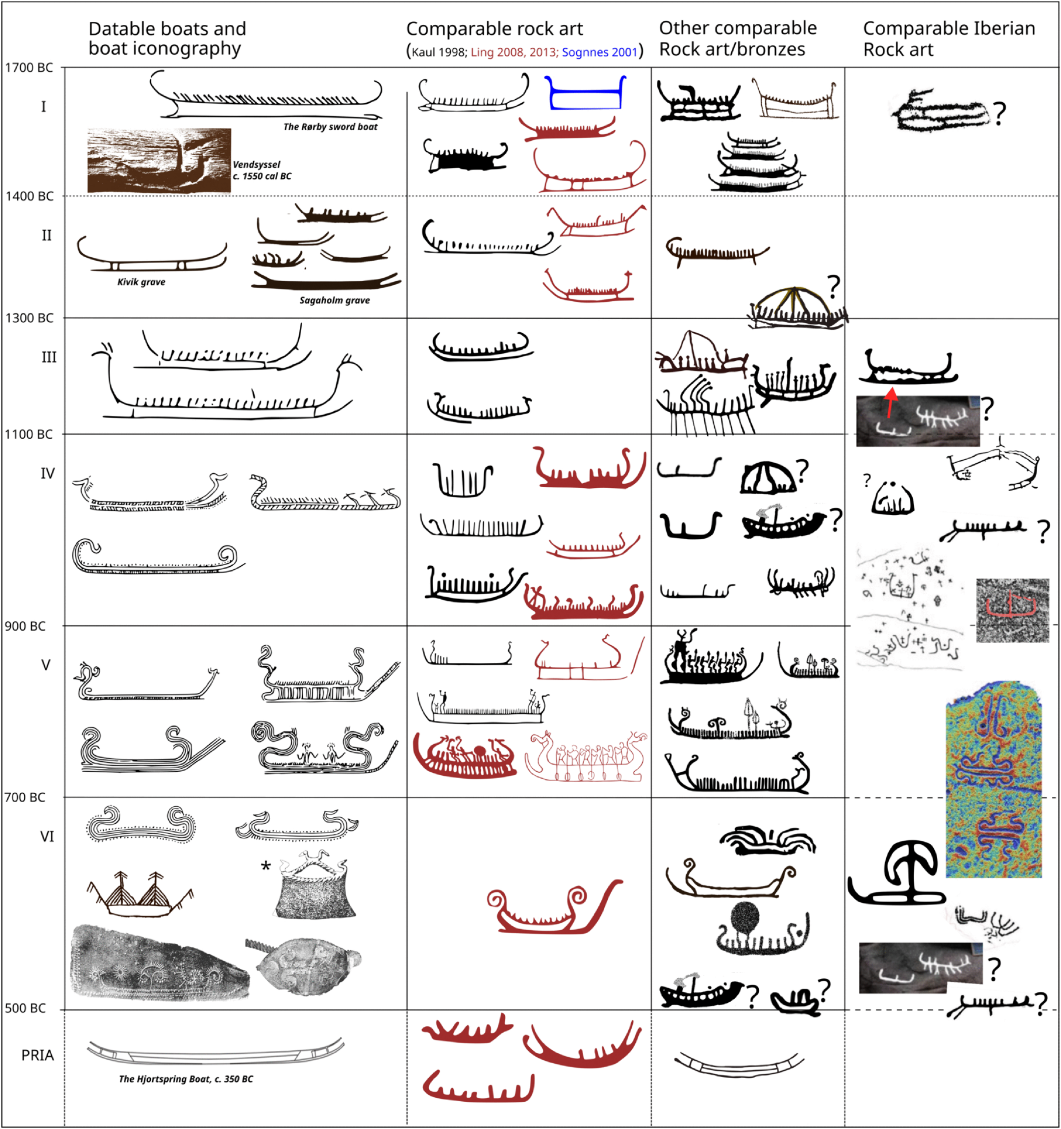
Overview of dated Scandinavian boat iconography adapted after Kaul [92,93], comparable dates for rock art according to Ling [98–99] and Sognnes [95], other comparable boat carvings from southern Scandinavia in relation to the Iberian rock carvings discussed in this article. Images with question marks signify boats for which the date is ambiguous. For example, in the larger boat from Järrestad the mast and yard could have been added at a later stage although when is impossible to tell – whether within the same year or a few hundred years later. High quality digital documentation of rock art boat representations can be found at the Swedish Rock Art Research Archives here: https://shfa.dh.gu.se/. Reprinted under a CC BY 4.0 license, with permission from Boel Bengtsson.

In Fig 4, we present an overview of how the Iberian iconography presented in this article might relate to the southern Scandinavian imagery. Following this general overview of the southern Scandinavian boat iconography, we now turn to the Iberian examples.

### Rock art digital recording

Formal methods are at the heart of our approach to rock art research [111], and this study is no exception. In this paper we present new digital documentation of boat depictions from northwest Iberia with the goal of addressing unresolved questions about their iconography and temporality through comparative typology (see above). Importantly, added to that, we also focus on various aspects of rock art making, such as the qualities of stone or the gestures of rock art making itself. Rock art production is a process of assembly in which many different components play a part in revealing meaning and temporality [112–114], and these are crucial to understand some of the key questions addressed through our research.

Four sites (Santo Adrião, Penedo do Muro, Auga dos Cebros and Borna) had previously been recorded using Structure from Motion (SfM) photogrammetry, but the resolution of the existing 3D models did not resolve remaining interpretative questions. Additionally, three more sites (Eira do Louvado, Laje da Churra, and Senhora de Encarnação) were recorded digitally for the first time. We employed the comprehensive digital methodology of the RAW project, which includes two types of 3D models produced using state-of-the-art laser scanning [115] and high-resolution SfM techniques [116] at four sites (Santo Adrião, Eira do Louvado, Penedo do Muro, and Auga dos Cebros), while Laje da Churra and Senhora de Encarnação were recorded photographically for SfM only.

Laser scanning was conducted using the Handyscan 700 red-light laser scanner, provided by the Swedish Rock Art Research Archives (SHFA), which captures approximately 480,000 measurement points per second and reproduces the scanned area with a resolution of 0.05 mm. Through interpolation, the highest actual output resolution is 0.2 mm [115]. SfM, an image-based modelling method, uses multiple static images and pixel recognition to generate three-dimensional point clouds [116]. For 3D reconstruction, we used the software Metashape. To enhance the visualization of the micro-topography of the 3D models and allow for detailed examination of surface detail in rock art, including marks of (re)carving, we used three additional techniques: Topography Visualisation Toolbox (TVT; https://tvt.dh.gu.se/), developed by researchers at SHFA, which is programmed to work with laser scans, a GIS protocol based on focal statistics in ArcGIS 10.6 [117–118], and Reflectance Transformation Imaging (RTI) [119–121]. TVT and focal statistics use depth maps, grayscale pictures of the 3D shape of a surface, where each pixel stores the distance between the sensor (or virtual camera) and the surface being recorded. TVT enhances carvings by analysing the geometry of 3D data: it uses depth maps to isolate small variations in surface curvature and computes surface normals, blending these with enhanced depth‑based outputs to reveal subtle topographic features. Focal statistics also relies on depth maps, but instead of calculating normals, it applies mathematical operations to local neighbourhoods, such as standard deviation, to accentuate micro‑scale height differences that correspond to carved motifs. RTI (Reflectance Transformation Imaging), by contrast, excels at photorealistic rendering of surface texture. The technique does not analyse surface geometry directly; instead, it captures how a surface reflects light from many directions and uses polynomial texture maps to reconstruct its shape and let the viewers “relight” the object interactively through easy-to-manipulate 2.5D models, revealing details that become visible under changing illumination [122–123]. We used the Highlight RTI capture method, which is cost-effective, mobile, and flexible, allowing for the recording of elements of variable size. The capture and processing of RTI datasets, including in rock art studies, are described in various sources [119–121,124,125]. The output can be analysed using the RTI Viewer [126], which allows for interactive visualization through dynamic re-lighting of the surface.

### GIS-based landscape analysis

The landscape placement of rock art sites is crucial for interpreting boat imagery, yet systematic GIS appraisal has only been conducted at one site with boat depictions in northwest Iberia. Traditionally, style in rock art studies has been used to interpret social metaphors, such as identity [127–128] and establish chronology through comparison (see above). Analysing the landscape alongside traditional style studies allows for deeper understanding of the use and value of rock art sites [129]. This builds on Ingold’s [130–131] concept of ‘taskscapes,’ which comprise cultural activities embedded within natural features. Rock art is associated with activities ranging from religious and ritual to acting as markers of the land, understood through surrounding landscapes [113,132].

Landscape analysis examines the physical location and morphology of the engraved outcrops, understanding both as symbolically important as the art itself [132]. Studying rock art surfaces and the wider landscape helps understand the creation of the art and its meaning, rather than viewing rock art as merely symbolically encoded [111,133,134]. GIS has been applied to landscape studies since the 1990s, focusing mainly on distribution patterns [135–136]. Visual characteristics have always been important in archaeology to understand site location and significance [136]. Viewshed analysis is a popular GIS technique for analysing rock art placement, environmental relationships, and visibility [137–139]. Ways of perceiving the landscape are deeply tied to our own culturally mediated ways of perceiving the world and modern concepts of topographic boundaries [140–141]. Thus, to approach the nature of past perceived landscapes, it is ideal to explore overlapping cues from visibility and mobility analyses [137,142–144].

This paper presents the results of a GIS-based landscape analysis of rock art sites featuring boat imagery in northwest Iberia. Our study explores landscape features governing site placement and their relationship with bodies of water. Until now, only Laje da Churra had been analysed using GIS tools [77]. We provide further empirical evidence to advance the interpretation of these images within a broader framework of late prehistoric connectivity. Various GIS tools were used for landscape analysis, such as slope, elevation, measurements to landscape features, individual viewshed, and cumulative viewshed [145].

The viewshed from a site varies greatly depending on the eye height of the viewer in comparison to the surrounding landscape. Whereas viewshed over land is often interrupted by trees and hills, it can vastly increase when facing the uninterrupted horizon of the sea, something not least seafarers seeking land would have been acutely aware of [146, Fig 6]. Most GIS based software used by archaeologists only allow for calculating a viewshed of 3 km, which does not consider the full effect of earth’s curvature and therefore loses potential links between a site and important landmarks. In relation to nautical rock art, it is the potential connection with waterways and the sea that might be lost. For calculating the maximum distance of viewsheds in clear weather from the sites presented here we are using a convenient on-line tool [147], which following some initial test calculations was found to be reasonably accurate. Following this, the calculations have been visualized using the viewshed analysis tool in ArcGIS Pro with the maximum distance being inputted as the Distance to Horizon. The viewsheds were calculated based on the 30 m resolution European Digital Terrain Models from Hengl et al. [148], using the Viewshed2 tool in ESRI ArcGIS Pro 3.5.0 [149]. The observer’s eye level height was taken to be a maximum of 1.75–1.80 m. GIS analysis is affected by vegetation cover, human vision, and potential DEM errors and resolution [136,144]. The results should be assessed cautiously, but understanding the relationship between sites and surrounding landscape features is crucial.

**Fig 5:**
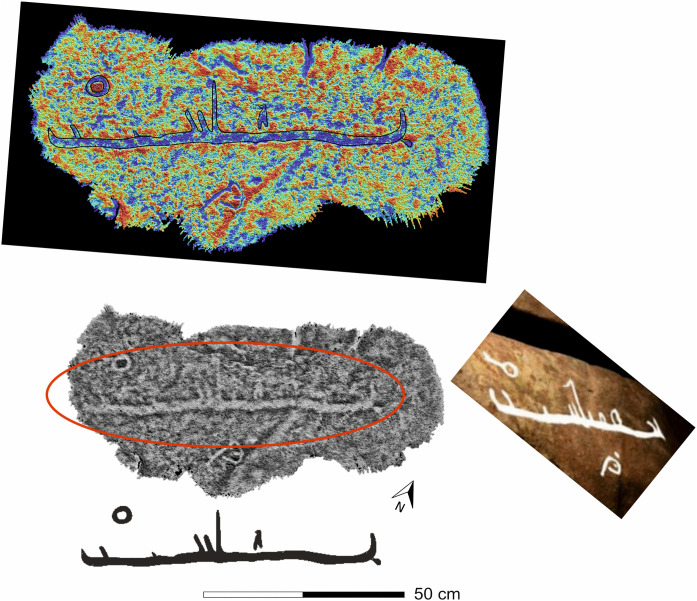
Boat 1 in panel 2 Santo Adrião. Enhanced visualization created from a high-resolution 3D scan. Low resolution SfM 3D model: https://skfb.ly/oupXD. Reprinted under a CC BY 4.0 license, with permission from Marta Díaz-Guardamino, Ashely Green and Boel Bengtsson.

### Rock art boat imagery in Northwest Iberia: A new assessment and comparison with Scandinavian images

This section presents a new evaluation of rock art boat depictions in each of the sites documented in northwest Iberia based on available records (new and existing), and their comparison with Scandinavian imagery. We address a series of unresolved but important questions about their iconography and temporality, including the typology of the vessels depicted, their dating and context within their respective panels.

### Santo Adrião

The site of Santo Adrião consists of eight engraved outcrops which were excavated and recorded in 2015 [2]. It is in the parish Âncora in the north of Portugal, which has a natural seashore, located 2,4 km away. The site sits on the western mid-slope of Monte do Cúturo, at an altitude of 223 m, with good visibility to the coast and mouth of Âncora river [2]. While the outcrops are currently surrounded by a dense coverage of an invasive species of tree, they are located on a very steep slope (16 degrees) from which there are views of the Afife and Âncora beaches and the Âncora river valley, very rich in alluvial tin (see above). A low-resolution 3D model of the site can be explored here: https://skfb.ly/oupXD.

**Fig 6:**
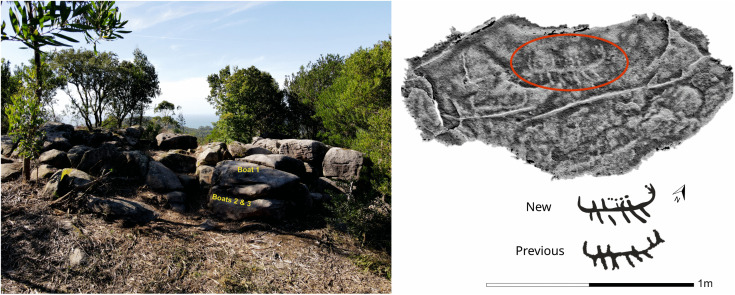
Boats 2 and 3 in panel 2 Santo Adrião. Left: Outcrop with boat depictions and view of the sea to the west. Right: Enhanced visualization created from a high-resolution 3D scan. Low resolution SfM 3D model: https://skfb.ly/oupXD. Reprinted under a CC BY 4.0 license, with permission from Marta Díaz-Guardamino, Ashely Green and Boel Bengtsson.

The outcrop Santo Adrião 2 is 2.10 m long and 1.7 m high and has three boat petroglyphs and a few circular designs around them (Fig 5,6). Boat 1 is on the top of the outcrop and is the largest; it has a flat bottom, linear and oval shapes which could be crew and/or tents, and a circular shape above which has been interpreted as an astronomical feature.

The second boat has 5–6 oars and a prominent prow and stern. The third boat is simpler and features a straight “keel” line. Each boat has a different design. Santos-Estevez and Bettencourt [1] interpret them as maestro and canoe types and link them to the outcrop as a ceremonial space, with the boats representing mythical journeys.

Within the thousands of boat images in southern Scandinavia there are an abundance of depictions that can be discussed in comparison to the three boats at Santo Adrião (marked as number 4 in the mind map in Fig 3). Whereas most of the Scandinavian boat carvings most likely depict plank-built vessels [102] belonging to the same boatbuilding tradition as the Hjortspring boat [150], these boats can be depicted in a multitude of ways and still be meant to convey the same type of vessel. Hence, they are sometimes depicted with slightly rounded hull shapes and sometimes with entirely flat hull shapes. Likewise, hulls can be outlined as single or double lines, the latter sometimes completely hollowed out, whereas in the bronze imagery these same boat types might be outlined with a multitude of lines (Fig 4). This highlights the significance of the Hjortspring boat since this single boat find has made it possible to interpret these depictions of boats as indeed belonging to the same tradition [146]. This of course does not mean that the Santo Adrião boats belong to this boat building tradition but the above remain valid points to bear in mind when discussing any types of boat imagery where reliable source material of comparable boats is lacking.

Regardless of what boat tradition the boats on the Santo Adrião site belong to, it is very likely Boat 2 on panel 2 is indeed depicting a rowed vessel. A direct parallel to how the picture is carved can be found at the Bottna 6 panel on the Swedish west coast (Fig 3), which appears to show a boat with four or five pairs of oars. Bottna 6 can in relation to established chronologies be attributed to period IV (c. 1100−900 BCE) [96: 87]. A slightly earlier date might be given to a boat at Tanum 95 which more clearly depicts a rowed vessel [96]. If assuming each oar represents two oarsmen seated on a thwart, the Tanum 95 boat most likely represents a boat that was c. 14 m long excluding any hull projections at the end ships [102]. If we assume a similar spacing between oarsmen, the Santo Adrião vessel (Boat 2) with its potentially six visible oars might have been 8 or 9 m long. Whether each oar represents a single crew, or a pair is not possible to determine.

**Fig 7:**
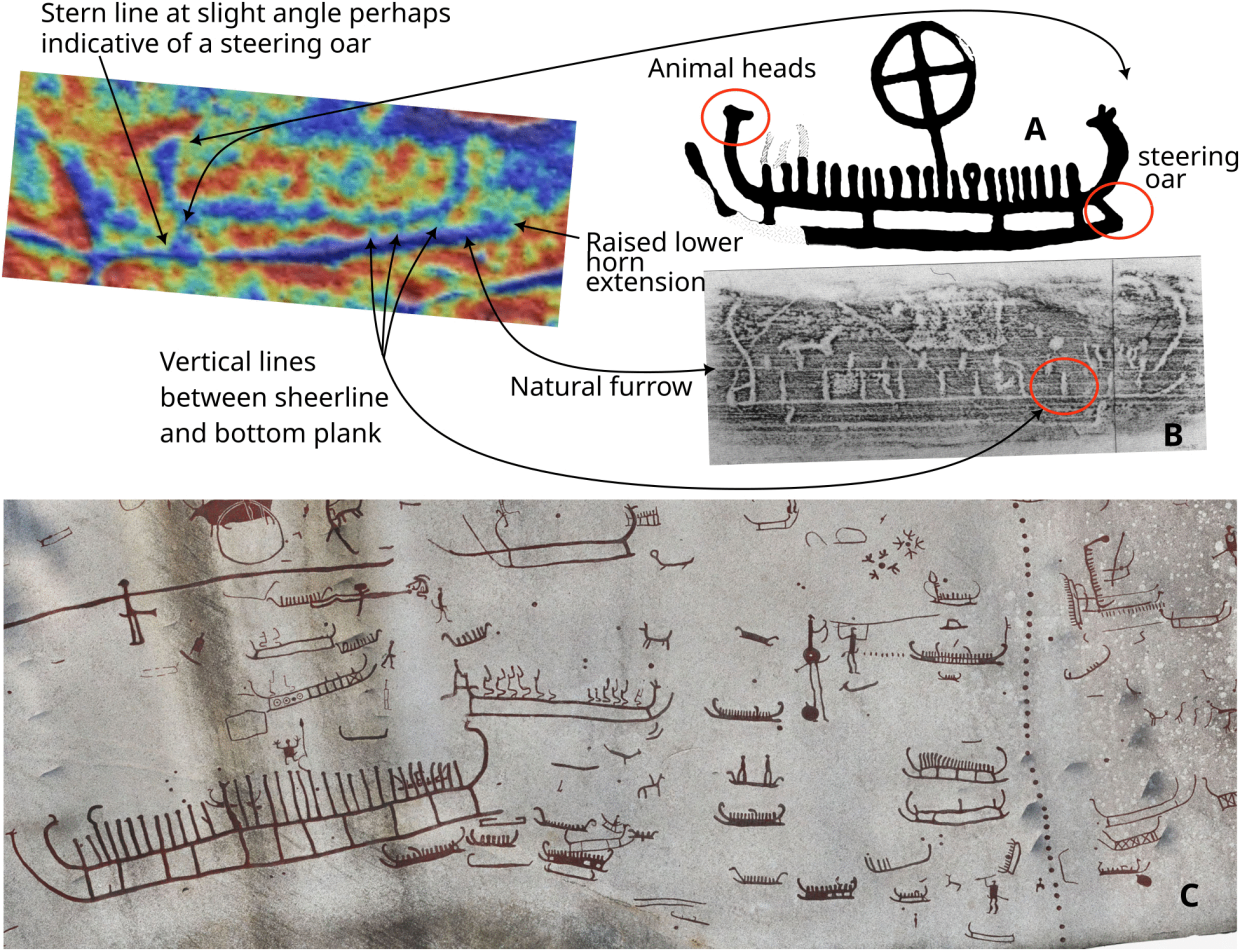
Boat 3 in panel 2 Santo Adrião with details which is suggestive of direct parallels from within the southern Scandinavian boat imagery. **A)** Boat from Bottna, west Sweden, **B)** Rubbing of boat from Himmelstalund, east Sweden, **C)** Part of a rock art panel from Tanum, west Sweden, after Stöltig [151], Bengtsson [93]; Horn & Potter [152]. Reprinted under a CC BY 4.0 license, with permission from Boel Bengtsson. High quality digital documentation of rock art boat representations can be found at the Swedish Rock Art Research Archives here: https://shfa.dh.gu.se/.

The new high-resolution scan of the Santo Adrião Boat 3 suggests an exciting new interpretation of this vessel as of a clear Scandinavian type (Fig 7). Because of the radical difference from previous interpretations and its potential implication of direct communication between Scandinavia and Santo Adrião, there is reason to take this new interpretation with caution. In Fig 7, the newly discovered details of Boat 3 are highlighted in comparison to two boats from Bottna and Himmelstalund in western and eastern Sweden (marked A and B), whereas the photo below shows the variability in how boats are depicted on individual panels within the region (C). The elements that suggest Boat 3 might depict a Scandinavian type vessel include; (1) the use of natural furrows in the composition of the boat, (2) vertical lines carved across the hull of the boat, in this case connecting the former single line hull with a natural furrow below, (3) a small upward projection has added to the natural furrow indicating the raise of the lower horn projection in the bow, (4) the use of a slight outward and downward slant in the aftmost of the vertical lines as if conveying a steering oar [153], and finally (5), the animal head/heads decorating the either one or both end-ships. Could this represent a Scandinavian vessel? If so, the shape of the end-ships would suggest it represent a boat typical of the Early Scandinavian Bronze Age, whereas the potential animal heads decorating the upper horn projections would suggest a date from period II or III (c. 1400 or 1300−1100 BC).

### Eira do Louvado

**Fig 8:**
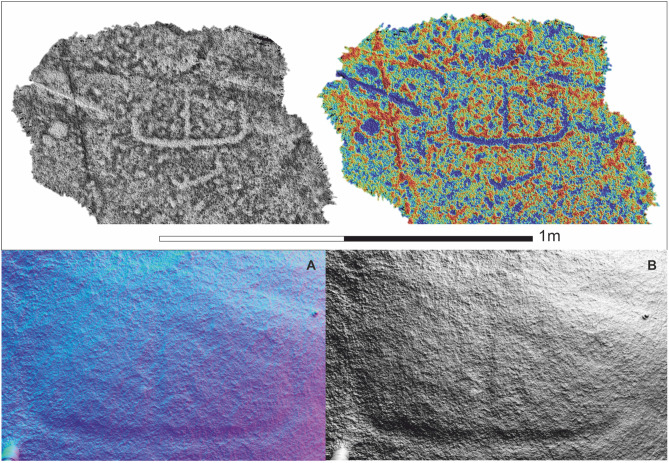
Enhanced visualizations created from HD 3D scans of the carvings of Eira do Louvado and RTI outputs (A. Normals, B. Diffuse). Reprinted under a CC BY 4.0 license, with permission from Marta Díaz-Guardamino and Ashely Green.

This open-air rock art site is in the parish of Carreço, Viana do Castelo, a coastal city in northern Portugal situated next to the estuary of the Lima River. The site was first presented by Bettencourt [154] and it is currently under investigation [18]. The outcrop where the carvings are placed is slightly raised from the ground on a gentle slope. The site (33 m elev.) is very close to the coast (1.2 km away). As noted above, the 3 km viewshed shows expansive views down approximately 5 km of the coast, which is straight with small irregularities and inlets, although in the 2nd and 1st millennium BCE the coast was more indented and lagoon-like [155]. (Fig 8).

Previous work [18] documented a boat with a mast and several cup marks around it which may be associated with the sun in Scandinavian mythology and Bronze Age Atlantic travellers and navigators. Recent 2.5D and 3D documentation at the site reveals a boat with a straight hull line curving abruptly to near vertical lines at either end (Fig 8). There is a central mast with a small dimple in the middle of the line. There are also various vertical lines or cup marks inside the boat that could be representing a crew. Two cup marks can be seen to the left of the boat and one below it. There are also possible lines connecting the mast and both ends of the boat, perhaps representing stays. Finally, there is a line below the main boat which may depict a smaller boat.

Again, there is very similar imagery on display within the southern Scandinavian rock art material. In Fig 3, the Eira do Louvado boat is marked as number 6, with several parallels both in relation to the straight single line hull shape and the more vertical end-ships (the latter present in Scandinavian rock art as both single, double or multiple lined hulls). Another important similarity is the single vertical line amidships of the vessel which is a common way of depicting a mast within the Scandinavian rock art boat imagery [93,96,103,156,157]. A direct parallel to this way of depicting a mast is found on a piece of pottery discovered during excavations in the city centre of Lisbon in 1995, on which a boat believed to be of Phoenician origin is depicted (see bottom left in Fig 3) [84]. Based on Scandinavian imagery, the Eira do Louvado boat or boats are different from those of Lisbon and might date from c. 1300−1100 BCE or slightly later (Fig 4).

### Laje da Churra

This large open-air rock art site has over 1200 engravings across 20 panels [76–77]. These carvings date from the Neolithic to the Iron Age and present a wide range of iconography [76]. The site is situated at the foot of the west hillside of Santa Luzia Mountain range, surrounded by fields of livestock and housing which cover part of the site. The panels are on a gentle 7-degree slope, with an elevation of 67 m. Laje da Churra is the only site in this work to have been analysed through GIS. Santos [77] assessed the visibility range of this site, concluding that the mountain range and sea create natural barriers around this site which have symbolic importance. The coast is just 1.5 km to the west and is highly visible from the site.

**Fig 9:**
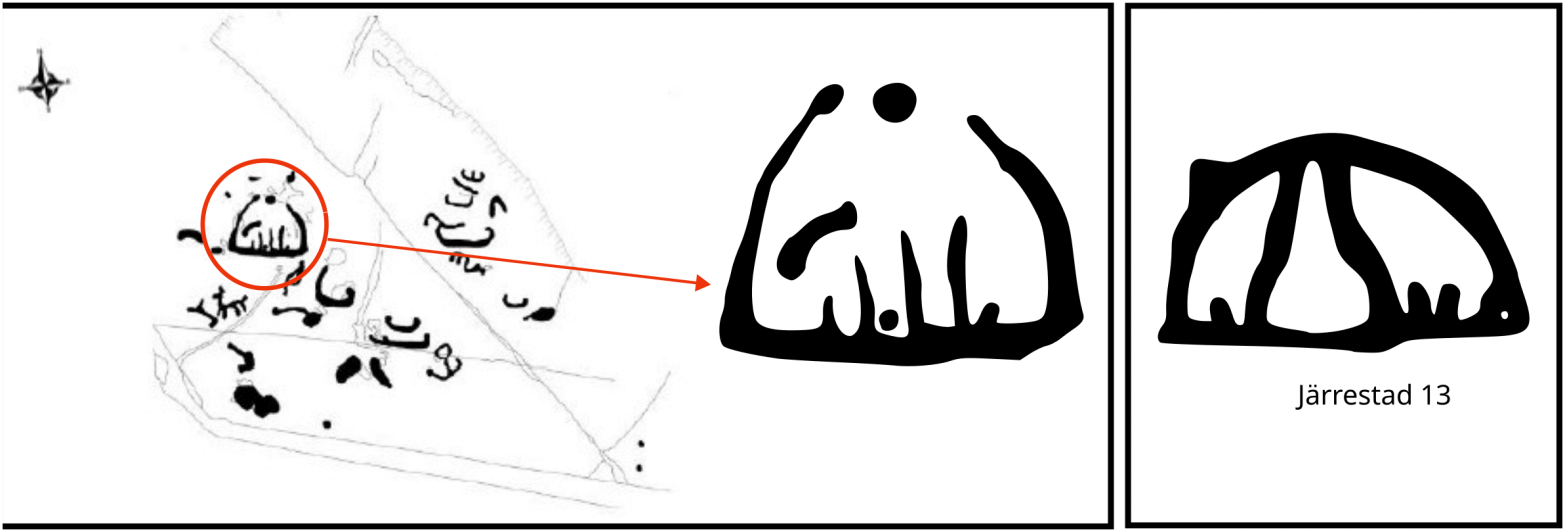
Panel 18 of Laje da Churra highlighting the ‘triangular boat’ petroglyph based on the interpretation by Santos [77], with additions. Reprinted under a CC BY 4.0 license, with permission from Boel Bengtsson.

**Fig 10:**
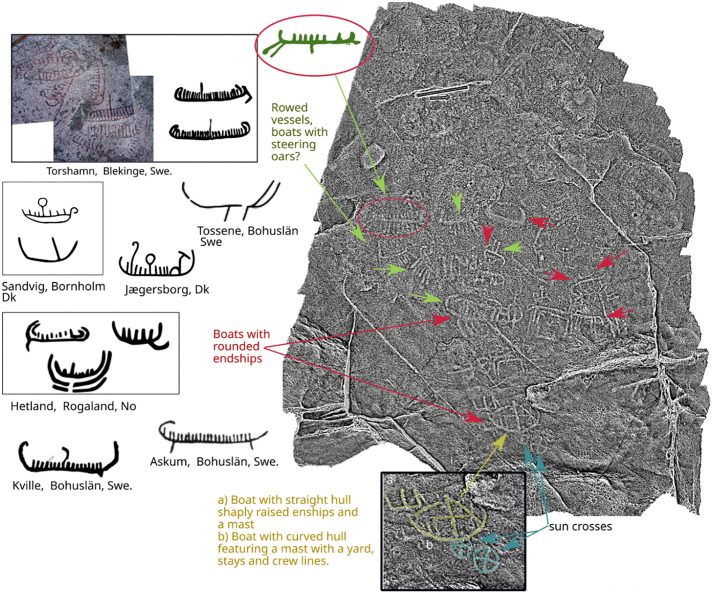
Panel 6 of Laje da Churra highlighting the boat iconography identified by Santos [76–77] in the southwest corner of the panel and a boat with mast and rigging, yard and curved hull at the bottom of the composition. Several new boat depictions were recently identified by Marinho, Bettencourt and Sampaio after the capture of new HD 3D documentation [21]. Green arrows point at boats with steering oars/oars, red arrows point out boats with rounded end-ships and the blue arrow point out the sun cross. Reprinted under a CC BY 4.0 license, with permission from Boel Bengtsson and Ashely Green.

On panels 6, 11, 16, 17 and 18 (Figs 9,10) boats are featured alongside zoomorphs, segmented circles and linear designs [21,77]. There is a boat with straight hull shape, curved ends and small vertical lines perhaps representing a crew, (Fig 10). The “triangular” boat (Fig 9) also has a straight hull line and upwards protruding crew lines, but also high-end ships and a central cup mark. Bettencourt [14–15] notes the stylisation of the boats with crew, oars, possible nets, and the connection to the cup marks which could represent the sun. Santos [15] shows that 66% of the boats at the site are single lined boats and 34% are “triangular boats”, but altogether they are only 2.22% of all motifs at this site. Photogrammetry carried out at panel 6 from Laje da Churra by Marinho, Bettencourt & Sampaio [21] made it possible to identify more boats, some of them with curved sterns and vertical lines representing a crew, and another boat with a curved hull line, a mast featuring what could be interpreted as a yard and stays (Fig 10).

Some Portuguese authors consider the hypothesis that the first representations of boats are from the Early Bronze Age, based on a possible halberd associated with a vessel, in Laje da Churra [77,85]. In support of this, several boat depictions at Laje da Churra appear to have clear parallels within Scandinavian boat imagery. The way in which steering oars are represented on several of these boats also have clear parallels within the Scandinavian material. The boat circled in red on panel 6 (Fig 10), seems to depict a single line boat with seven crew lines. In addition to crew lines, the boat features two longer lines protruding backwards from the stern, another one roughly amidships, and finally a likely steering oar in the bow – in all potentially four steering devices. The variability in how these steering devices are mounted most likely reflect oars being moved around and secured depending on where they were needed. Examples of how steering devices are depicted within the Scandinavian rock art are highlighted with red circles in Fig 3, to the right of the Iberian boats numbered 3 and 4. This material also appears to include potential side rudders [96].

Panel 6 features various other boat carvings, several of which have faint lines suggesting they represent rowed vessels. Rowed vessels are not common within the Scandinavian rock art with perhaps two dating to the Bronze Age [96: 81,87] (from around 1300 BC). The panel also includes two masted vessels – one of which has the same straight bottom plank as the masted boat from Eira do Louvado. The other, in contrast, has a very rounded hull, several crew lines, and its mast is fitted with a yard and supported by stays. Exactly what type of boat this curved vessel might represent is difficult to know. Similar hull shapes can be found in, e.g., Rogaland, Norway (Fig 10), but it might equally represent a masted vessel from other parts of the Atlantic coast. A tempting interpretation is that it might represent a sailing curragh which is a boat type known to have been used in the Atlantic tin trade in the 6^th^ and 4th centuries BC [158]. Such vessels are believed to date back into at least the Neolithic period if not earlier [159]. Such an older date might be supported by the sun-cross which is overlaying the hull of this boat. In Scandinavian rock art sun crosses are very common and often found in combination with boats, sometimes even mounted inside boats (see the boat from Bottna in Fig 7) [21]. A recent review has revealed that sun crosses (also called ‘segmented circles’ in Portugal), are relatively common in NW Iberia with more than 100 depictions across twenty-nine rock art sites [21]. The combination of boats and sun-crosses in the Scandinavian material might be set in relation to the importance of the sun for navigation [160].

### Senhora de Encarnação 1

The outcrop of Senhora de Encarnação 1 (Lovelhe, Vila Nova de Cerveira, Viana do Castelo), is in the left bank of the Minho River, on a small intermediate platform on the western slope of Serra da Gávea, at about 225 meters above sea level, with visibility of the Atlantic Ocean, and the river mouth and estuary of the Minho.

**Fig 11:**
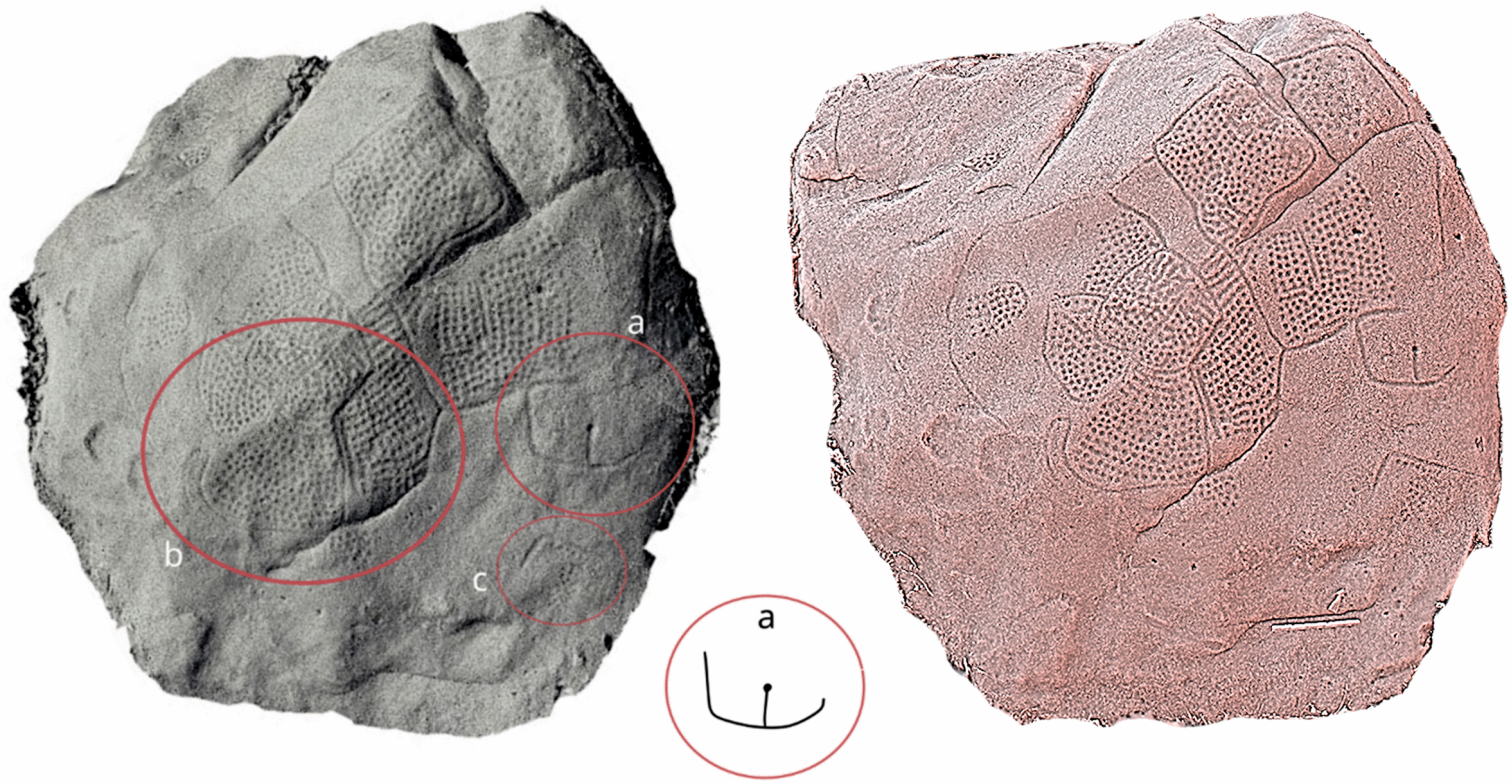
Enhanced visualizations of the 3D model of the outcrop with rock art of Senhora de Encarnação 1. Three potential boats are circled on the left-hand visualization. Reprinted under a CC BY 4.0 license, with permission from Diogo Marinho and Boel Bengtsson. 3D model can be downloaded here: https://skfb.ly/pHyKA.

The granite outcrop is elevated from the ground and has arched shape. Rock art motifs are found at the top and on the southwest (rare), south and southeast slopes [161–162]. The boat depictions are located on the southeast slope (Fig 11). The first boat motif (a in Fig 10) measures c. 47 cm in length (measured on the hull) and about 58 cm from bow to stern, which are asymmetrical. The hull is defined by a horizontal groove. It possibly includes a mast with a small depression on top. The boat is orientated northeast – southwest. The second boat figure (b) shares hull traits with the first boat but is larger. It is orientated east-northeast to southwest-west and features at least a bow or stern. Its interior includes small depressions parallel to the hull, plus a set of small depressions and grooves perpendicular to it. The bow or stern is adjacent to a faint circular motif (concentric circle), possibly older than the boat composition.

At the start of the southern slope, there is the carving of half a boat (c) with a curved bow or stern. The large concentration of small depressions (cup marks) appears to be contemporaneous with the boat carvings, given how they interconnect and complement each other on the rock surface.

Although the three boat like carvings appear either as part of or near intricate compositions of lines forming square like shapes filled with depressions, cupmarks and grooves, the hull like shapes appear to stand out from the overall composition and are of a similar type to other, more clearly depicted boat imagery in the region. Thus, the possibility that they are attempts to depict boats cannot be excluded. If boat ‘a’ is indeed featuring a mast, it might date to a similar period as the boat at Eira do Louvado (Fig 8).

### Penedo do Muro 1 and 2

These are two panels located 20 m apart, in Sandín, Monterrei, south Galicia [3]. The sites are in the uplands, at an elevation of around 800 m and more than 100 km from the coast. The panels sit on gentle slopes (5 and 7 degrees) at points in the landscape from which one can see the main creeks of the area, Bocas do Muiño and Carzoá de Barroca, where they join the river Montes (within 3 km visibility range).

**Fig 12:**
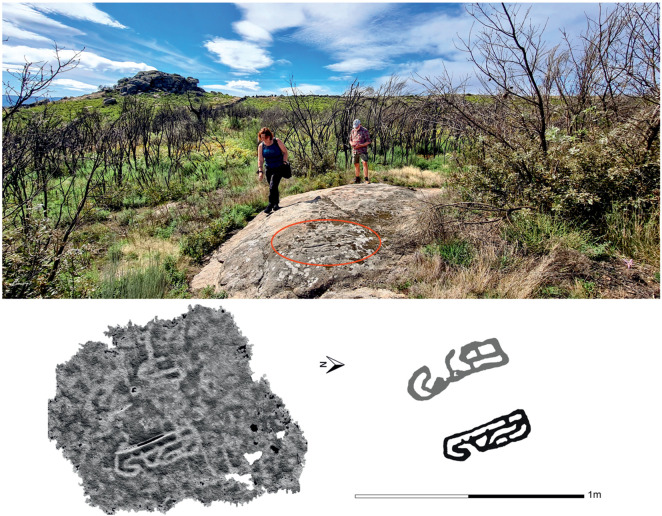
Landscape setting (top), 3D visualization of the Penedo do Muro 1 boat petroglyph (bottom left) and tentative outline of its inverted twin (bottom right). 3D model: https://skfb.ly/oAyTP. Reprinted under a CC BY 4.0 license, with permission from Marta Díaz-Guardamino and Ashely Green.

Overall, this rock art ensemble includes three flat outcrops and evidence of three movable elements which are included as part of a later dry-stone wall [163]. From these, Penedo do Muro 1 and 2 feature potential boat petroglyphs alongside cup marks, circles, linear carvings and podomorphs [3]. There is a possible boat in Penedo do Muro 1, made from a series of interconnected rows, that also has an inverted twin, as revealed by the most recent 3D scanning work (Fig 12).

**Fig 13:**
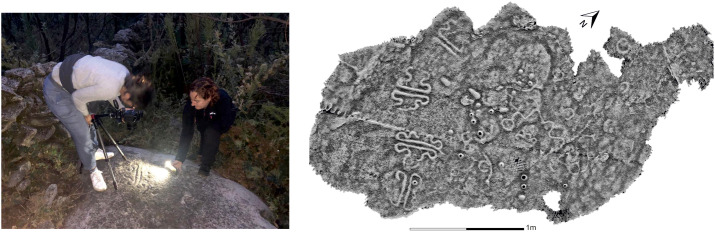
RTI capture (left) and 3D visualization (right) of the Penedo do Muro 2 panel. (3D model: https://skfb.ly/oAArG). Reprinted under a CC BY 4.0 license, with permission from Johan Ling, Marta Díaz-Guardamino and Ashely Green.

**Fig 14:**
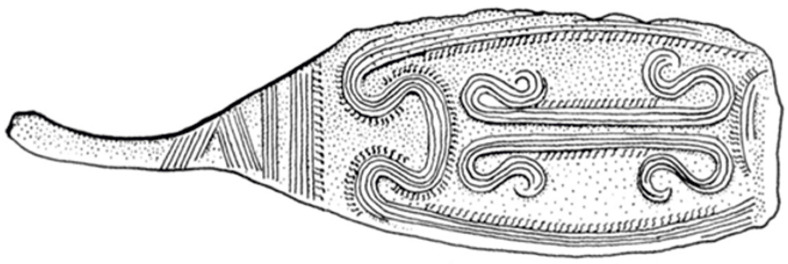
Ships depicted on a Late Bronze Age razor. Reprinted from [92] under a CC BY 4.0 license, with permission from Fleming Kaul, original copyright 1998.

Penedo do Muro 2 has possible boat figures on the right side made of serpentine shapes. Initially interpreted as boats [163], these motifs were later re-interpret as linear paths and asymmetrical ‘keys’ [3]. Digital visualizations generated from new high resolution 3D models within the RAW project revealed a clearer outline of the series of motifs distributed across the panel, as well as a neat outline of the boat-like figures, which also have inverted twins, very much like those known from the Scandinavian Bronze Age iconographic tradition (Figs 3,13,14) [164]. Medium resolution of both models can be seen as partial 3D models on Sketchfab (https://skfb.ly/oAArG).

Indeed, the clearest parallels here is this ‘keel to keel’ position of boats with s-shaped end ships, which in Scandinavian material is commonly featured on neck rings dated to period VI, c. 700−500 BCE (Fig 3: 4, Fig 14). The same s-shape also appears on the end ships of boats in the rock art with Tanum 325 as a prime example (Fig 3). Thus, a direct comparison with the Scandinavian material would suggest the Iberian iconography date to between 900−500 BCE (Fig 4). As for the Penedo do Muro 1 boat carving, this is harder to decipher, but possible parallels within the Scandinavian material are still evident, with two examples in comparison to which a date within the Scandinavian BA might be assumed (Fig 3).

### Auga dos Cebros

**Fig 15:**
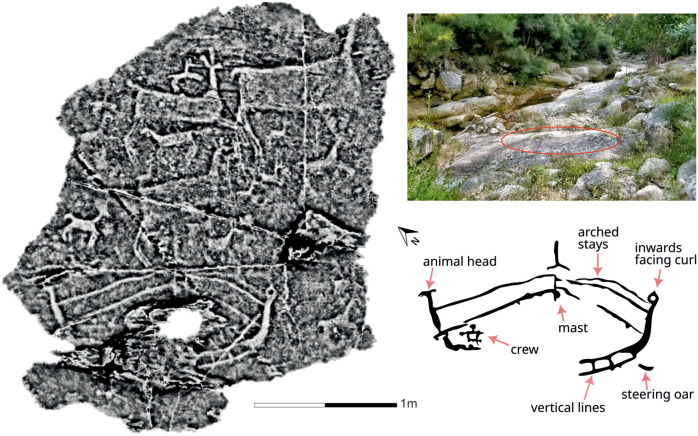
Boat 1 of Laxe Auga dos Cebros. Left: 3D visualizations from the RAW project. Top right: landscape setting of the panel. Bottom right: interpretation of boat 1. 3D model: https://skfb.ly/pqNSX. Reprinted under a CC BY 4.0 license, with permission from Marta Díaz-Guardamino, Ashely Green, and Boel Bengtsson.

Laxe Auga dos Cebros is an open-air rock art site with 3 panels with boat depictions in the parish of San Mamede de Pedornes, located at the foot of the Villar River, 1 km from the cove of Oia [71–73,165]. The panels are almost flat and, when the Villar River is high, the water skirts the edge of the panels. Due to the stream, some fluvial erosion has occurred, alongside destruction to panel 1 from treasure hunters [166]. The sites are at 126 m elevation, on a gentle slope, mid-way down to the coast (Fig 15), with an ample view covering the land and the bay of Oia to the west (see below).

From the three panels depicting boats at this site, Auga dos Cebros 1 (Fig 15) is the most notable due to its size and complexity. The panel presents a series of quadrupeds all facing southeast except one facing northwest and a boat with potential human figures in the central area [71,72,165]. This seems to be confirmed by our recent 3D documentation at the site, while also revealing new details, such as new quadrupeds and a more detailed outline of the already known ones (Fig 15). The boat has a bow and stern, as well as a mast and rigging [79,165,167]. Mederos [165] observes the curved hull as being suitable for sailing in the Atlantic Ocean, basing this assumption primarily on the assumed hull shape of the late 14th c. BCE Uluburun shipwreck, discovered off the Turkish Mediterranean coast [168]. He further notes similarities between Auga dos Cebros 1 and Hellenistic and Cypriot boat iconography dated to between c. 1375–1150 BC [165]. This Mediterranean boat iconography clearly depicts different hull designs to that of Auga dos Cebros 1 and any vertical lines across their hull differs by being more closely spaced together and extending upwards from a ‘keel’-line that appears relatively thicker (compare Figs 10–12 in [165]). However, the way the rig is depicted, with two lines running from different heights at the top of the mast to the boat’s endships, the upper one appearing slightly arched, and even a third slightly arched line extending from the mast towards the stern, provide a clearer if not exact parallel to the Mediterranean boat iconography. Here, the Helladic pyxis from tomb 11 from Tageana shows the clearest parallel with three arched lines extending aft but only one forwards, whereas the boat depicted on the larnax of the Skapiera tomb shows three straight connecting lines between the rig and both end-ships.

**Fig 16:**
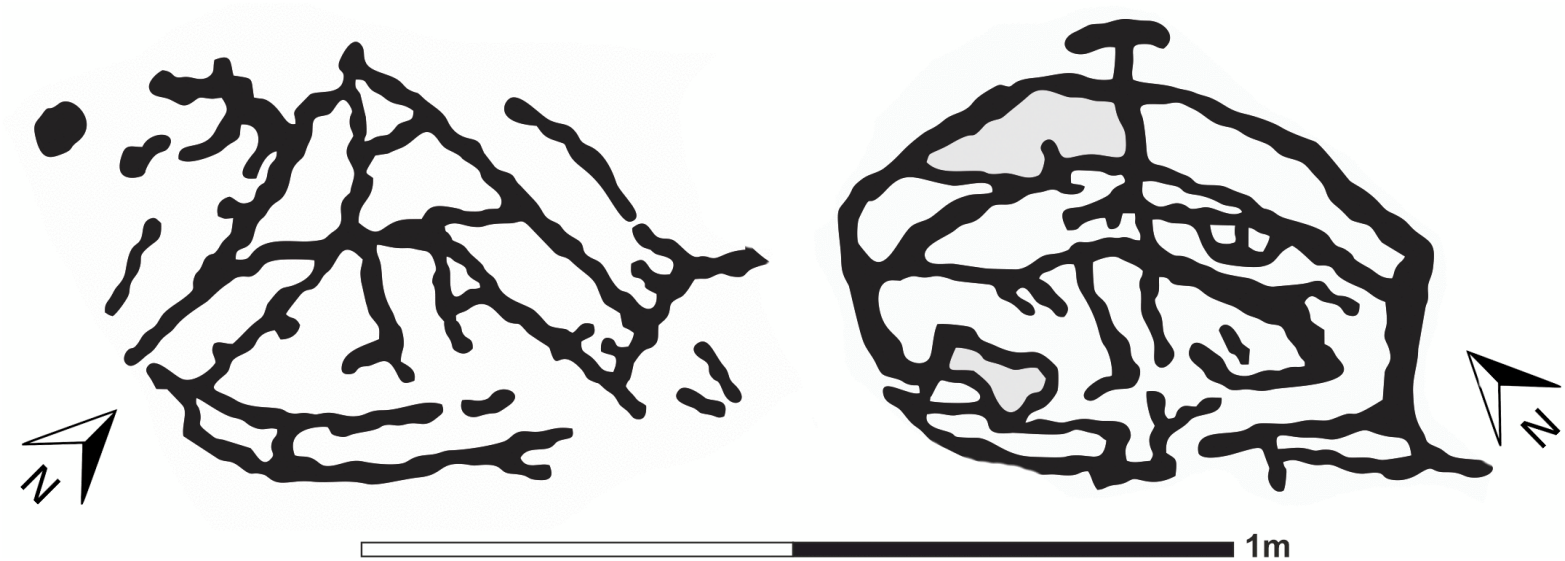
Auga dos Cebros boats 2 (left) and 3 (right) based on the interpretation by A. de la Peña Santos [73]. Reprinted under a CC BY 4.0 license, with permission from Marta Díaz-Guardamino.

Boat 2 and 3 (Fig 16) are just upstream from the main panel; they are smaller and simpler in design. Boat 2 (0.69 m) is interpreted as a single masted sailing vessel [79] and as a 13^th^/12^th^ century BCE Aegean boat due to the curved stern [169]. Boat 3 (0.55 m by 0.58 m) has a hard-to-interpret, overlapping design and was discovered in 2006 due to heavy rain [165]. These boats are interpreted as reed boats and plank boats, Aegean ships and eastern Mediterranean boats, highlighting the presence of Mediterranean and Atlantic trade links in the Bronze Age in Northwest Iberia [71,73,79,165,166].

**Fig 17:**
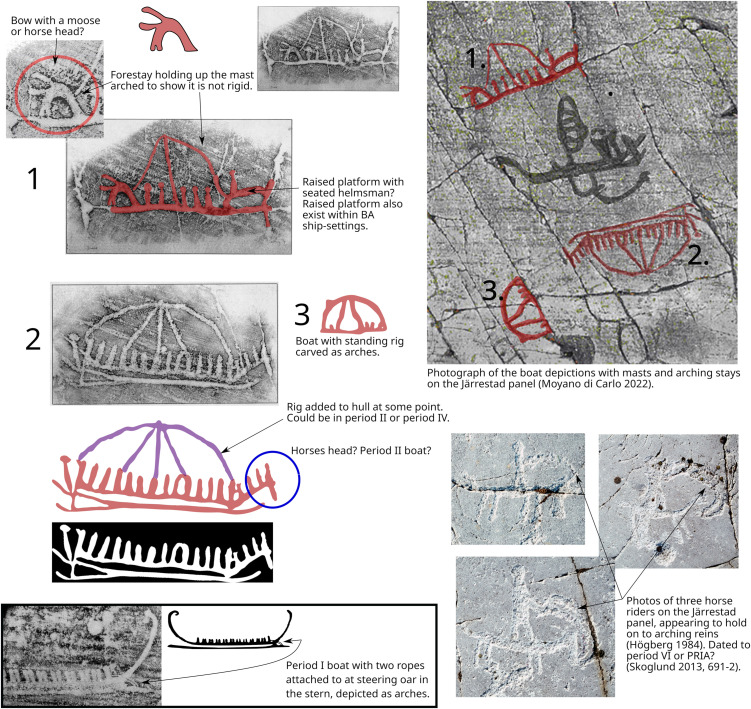
Overview of boats with rigs and stays marked in red on the top right as carved on the Järrestad 13 rock art panel in southern Sweden. The rock art panel is believed to have been used during two main periods, c. 1700−1100 BC and c. 900−200 BC [94]. Photos: Top right, reprinted from Julián Moyano Di Carlo [170] under a CC BY 4.0 license. Bottom right, Högberg [171]. Reprinted under a CC BY 4.0 license, with permission from Boel Bengtsson. High quality digital documentation of rock art boat representations can be found at the Swedish Rock Art Research Archives here: https://shfa.dh.gu.se/.

Of these three boat images, Auga dos Cebros 1 is notable for its clear similarity with a boat depiction at the Järrestad 13 site in southern Sweden, a site at which a further three boats that could be interpreted as having attributes suggesting masts, rigging and even sail can be found (Fig 17) [93,103,110]. In addition, Auga dos Cebros 3, might offer similarities depending on which way the boat is oriented. At present it is not clear what is ‘up’ and what is ‘down’ and the figure remains boat-like from either perspective (see Fig 3, where both perspectives are presented). From one these two angles this boat seems to share arched stays and a mast with boat 1 (Fig 4), something that has previously been suggested by Mederos [165] and Costas Goberna and de la Peña [172]). Altogether three of the boat depictions at Järrestad 13 have clear similarities with the Iberian material (Fig 17; see number 8 in Fig 3, next to the Scandinavian imagery).

In addition, boat carvings that offer similarities to the boats at Auga dos Cebros can be found at sites such as Svenneby 17 and Bottna 80 (Fig 3), both located on the Swedish west coast. All in all, similarities between these more complex Iberian boat(s) and boat imagery within the Scandinavian rock art include the presence of boats with arched stays, centrally located masts and/or a horse’s head featuring as a decoration in the bow and an inward facing curl in the stern. Parallels between the Scandinavian and Iberian boat imagery in question also include the date, with the Scandinavian boat imagery likely to belong to period II (c. 1300−1100 BCE) and/or period V (c. 900−700 BCE) (Fig 17); the same as has been suggested for the Auga dos Cebros boats.

Details that are interesting to note on the Järrestad 13 panel is that the arched rig and mast on boat 2 (Fig 17) could have been added at any time after the period II hull was initially carved. The carving of ropes as arches is frequently used to depict the reins of horses in Scandinavian rock art with several examples on the Järrestad 13 panel where they have been dated to period VI or later [94]. However, this way of depicting non-rigid details as arches is used already in period I in the wider region as indicated by a boat from Tanum (238) on the Swedish west coast on which such arches are used to depict the ropes attaching a steering device [96: 82-4]. In addition to arches used already in period I, the Vendsyssel carving would suggest that sail was used in Scandinavia in period II. Therefore, a period II date for the whole boat 2 composition (boat, mast and rig) on the Järrestad 13 panel cannot be excluded. In addition to masts and rigs, both boats 1 and 2 on this panel appear to depict some sort of raised platform in the stern, a detail also noted in some boat shaped stone monuments, so called ship-settings dated to the Bronze Age [173–174].

Both boats 1 and 2 at Auga dos Cebros have features suggesting a steering oar mounted on the side of the stern. Similar features appear on the Scandinavian rock art boats at Bottna 80 and Svenneby 17. These features might suggest that steering oars mounted on the side were used alongside two steering oars, one mounted at each end of a vessel, which is usually associated with Scandinavian boats of the period, with direct parallels between rock art and the Hjortspring boat [175].

The boat carving at Bottna 80 is extremely interesting since it appears to depict a similarly large seagoing vessel as suggested for the Auga do Cebros boats, perhaps even suitable for potentially direct sea journeys along the entire European Atlantic façade. The presence of such large vessels, perhaps 25 m long or more, has been suggested by investigations of paired ‘crew lines’ on Scandinavian rock art boats in relation to the Hjortspring boat and boat shaped stone monuments, so-called ship-settings, dated to the Bronze Age in Scandinavia [102]. Although the question of sail before the 6^th^ or 7^th^ centuries AD remains controversial in Scandinavia, the mounting evidence of potentially direct communication from Scandinavia to areas where sail was used during the Bronze Age is increasingly difficult to ignore, as is the imagery of boats with mast- and sail-like features and the location of centrally located mast- or postholes in large ship-settings dated from c. 1100 BCE [102].

### Alto das Veigas 2

**Fig 18:**
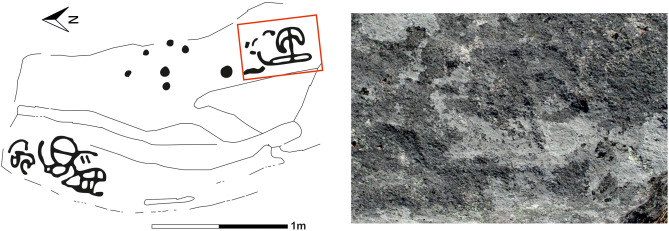
Line drawing and photo of petroglyphs on panel 1B of Alto das Veigas 2 based on the interpretation by Andrés & Costas [78]. Reprinted under a CC BY 4.0 license, with permission from Marta Díaz-Guardamino.

Alto das Veigas 2 is an open-air rock art site composed of two panels located in Mougás, Oia (Pontevedra) [78]. The site is situated on a gentle slope, at 461m elevation, with steeper slopes to the west and south to the coast. Its 1 km visibility range covers the river Mougás, which is clearly visible from the site, and at a range of 3 km the site has visuals to the sea, over a bay, west of Laxes. Panel 1B is in the south-west of the granite outcrop and is a sloping rock face, making it very easily visible. There is a boat depiction with a rectangular body and flat bottom (Fig 18) [78]. It has a small line going upwards which has been interpreted as the stern (and related to Aegean stylised narrow hull boats, called ‘frying pan ships’) and a mast in the centre with a half-moon sail [78].

**Fig 19:**
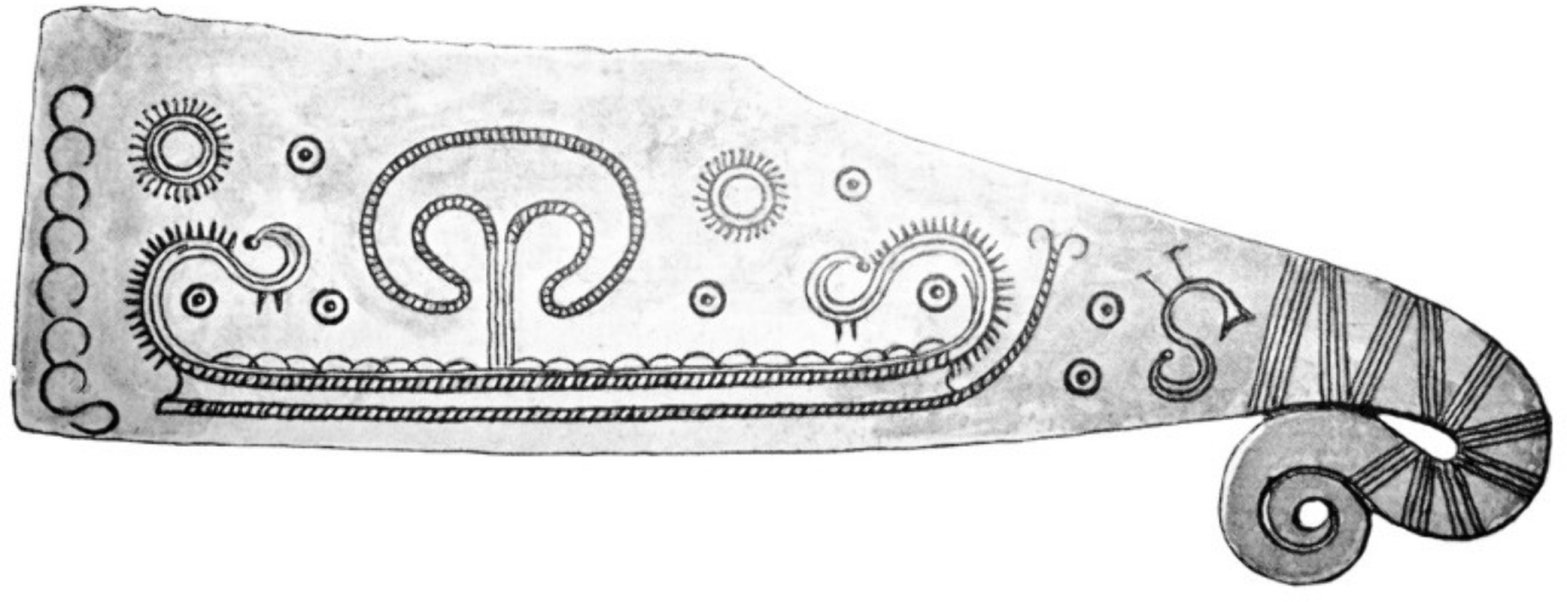
Ship, sailing towards the right, with solar images made on a razor from Honum, East Jutland, Denmark, c. 800 BC. Reprinted from [176] under a CC BY 4.0 license, with permission from Fleming Kaul, original copyright 2018.

Mushroom-shaped symbols on ships are known on Late Bronze Age razors from Scandinavia (Fig 19) [176]. As these are almost always depicted boats travelling to the right, Kaul [176] suggested that in LBA Scandinavia these boat depictions were related to the day- time voyage of the sun. However, as pointed out further down in the text, there is at least one example on the bronzes of such a boat facing left, just like this Iberian example. Another plausible interpretation of these mushroom-like figures is that they represent ceremonial axes or ‘cult’ axes [176]. Indeed, whereas most mushroom-like figures appear on razors, both amidships and as decorations on the end-ships of late Bronze Age boat depictions, and as a decorative feature on a similarly dated knife handle, the potentially corresponding shape within the rock art material is slightly different (see Fig 3). When comparing both variations to a ceremonial axe from Galstad in western Sweden dated to the late Scandinavian Bronze Age, its resemblance to both ‘mushroom’ and ‘cult axe’ is evident (Fig 3). It should be added that the mushroom shape on the Scandinavian razors has also been interpreted as a form of sail [177]. Thus, it might represent a complex symbol perhaps implying the connection between a cult object (axe) and the use of a boat with sail needed to acquire it, whereas the connection with the sun (the razors) might imply the use of the sun for navigation.

What is clear is that this type of attribute within both Scandinavian bronze and rock art material would suggest such details most likely belong to the same period which based on the bronzes provides a date of c. 900−500 BC (Fig 4). The appearance of the same type of attribute on a boat within an Iberian context with a hull shape featuring what might possibly be an extension from the lower sheerline, might suggest direct contact between the two regions at this time (unless the same types of attributes were known from other nearby regions). The difference between the Iberian depiction and the Scandinavian imagery is that the Scandinavian equivalent imagery only features on bronzes. Both the Iberian depiction and at least one of the Scandinavian bronze depictions travel to the left rather than the usual “right” (see the Haderslev razor in Fig 3).

### Escada 2

This recently published open-air rock art site [178] is composed by a large panel (5 m x 3 m) that is flat but raised in the centre, where most of the petroglyphs are found. The outcrop is located mid-way on the southwestern slope of Monte Faro de Domaio, 2.09 km north of the Atlantic coast, from which it has commanding views to the southwest (see below).

**Fig 20:**
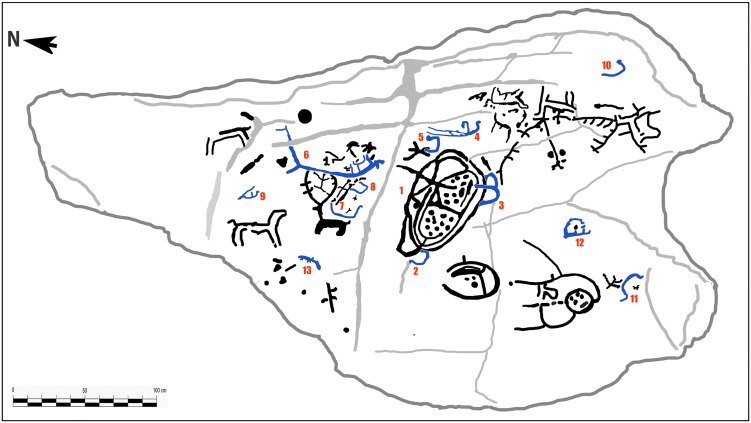
Escada 2 panel with boats highlighted in blue and numbered. Reprinted from [178] under a CC BY 4.0 license, with permission from Antonio Costa, original copyright 2023.

The panel features quadrupeds and boats (Fig 20). The possible boat in the centre (Fig 20: 1) has been described as having a central mast, straight hull line ending in high curved end ships [178]. There are cupmarks in the centre of the boat, which Costa [178] compares to 7^th^ century BCE Cypriot ceramics portraying boats and their cargo inside of the outer lines. The other boats on the panel are mixed in design, with both curved and straight hull shapes (2, 5, 10 and 11), some of which have end ships featuring an s-shape offering a direct parallel to many Scandinavian carvings (4 and 6).

**Fig 21:**
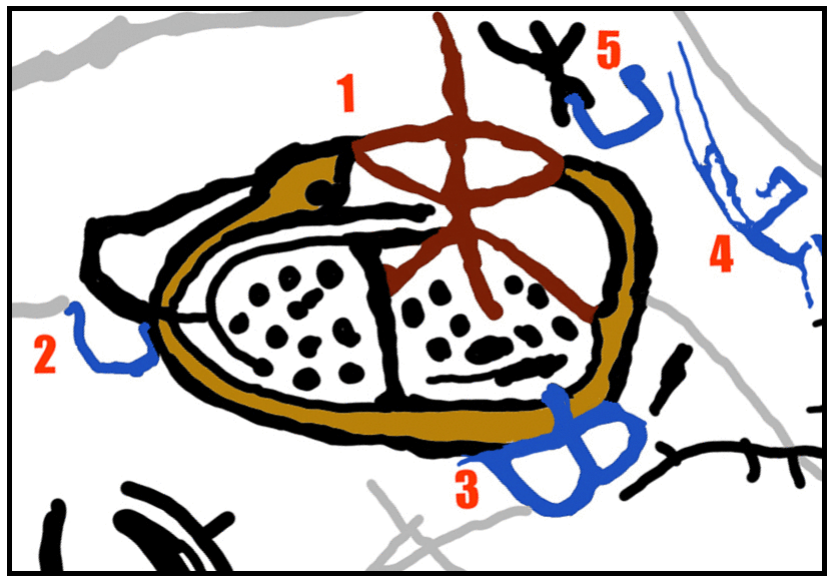
Escada 2 central figure interpreted as a boat, with colour added to highlight the outlines of the boats. Reprinted from [178] under a CC BY 4.0 license, with permission from Antonio Costa, original copyright 2023.

The Escada 2 boat figure is difficult to interpret in relation to the Scandinavian rock art material. Instead, it is the little boats to the left and top right of this main boat (Fig 21: 2, 5) that might be of interest since they are very similar to other boats already discussed in this article that appear on sites at Santo Adrião, Eira do Louvado, Borna, and Laje da Churra. Therefore, these small boats might date to a similar period of c. 1000−800 BCE (Fig 4).

### Borna

**Fig 22:**
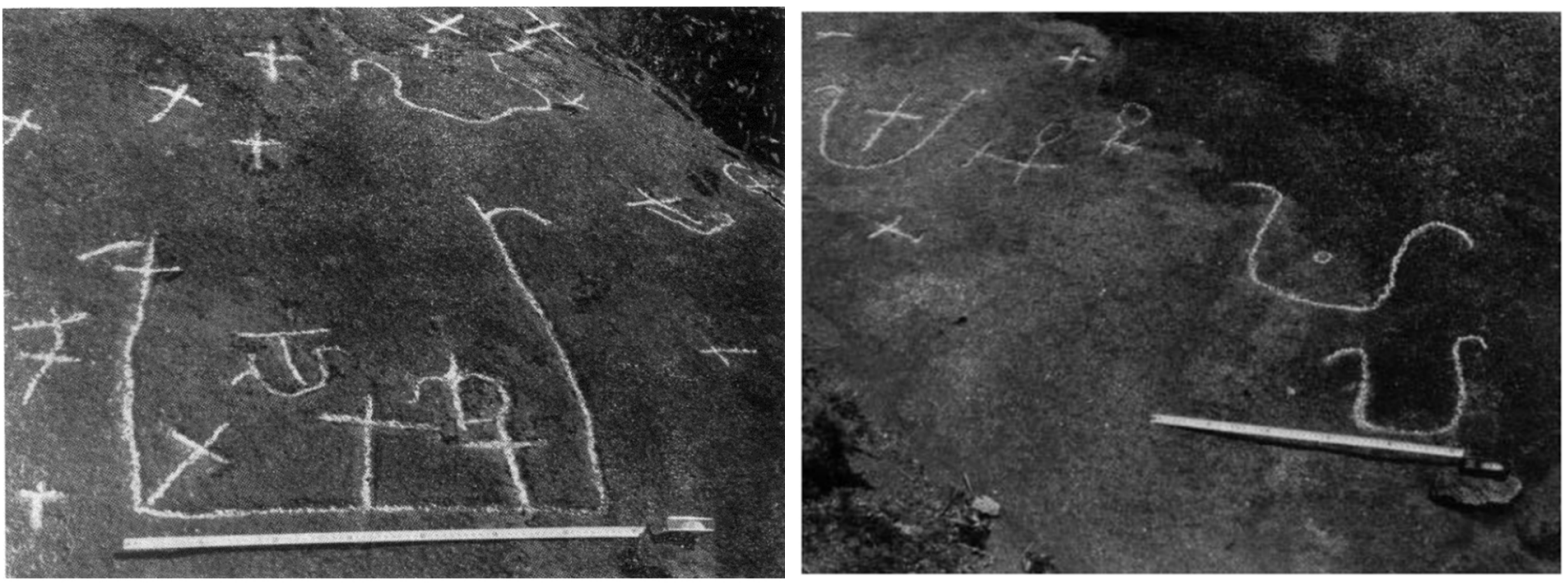
Nautical iconography from Borna. Reprinted from [179] under a CC BY 4.0 license, with permission from Fernando Alonso Romero, original copyright 1974.

The nautical iconography at the site of Borna is probably one of the most famous in the northwest of Iberia [79]. The main group of petroglyphs is featured in a very strategic position in the landscape. The site sits on a steep slope (22 deg.), only 0.32 km away from the coast, with an elevation of 102 m. The coastline here is very steep and not very accessible. The site has commanding views across the Ria de Vigo and opposite coastline (Fig 22).

The panel is quite large (12 m x 4 m), with thirteen boats displayed on the eastern face (the largest being 67 cm) [180], and one boat and human figures in the western face (Fig 22). The most recent photogrammetric documentation, nonetheless, has revealed up to 70 new boats [181] (see also 3D model by ‘arqueoestela’ here: https://skfb.ly/6SOIX).

**Fig 23:**
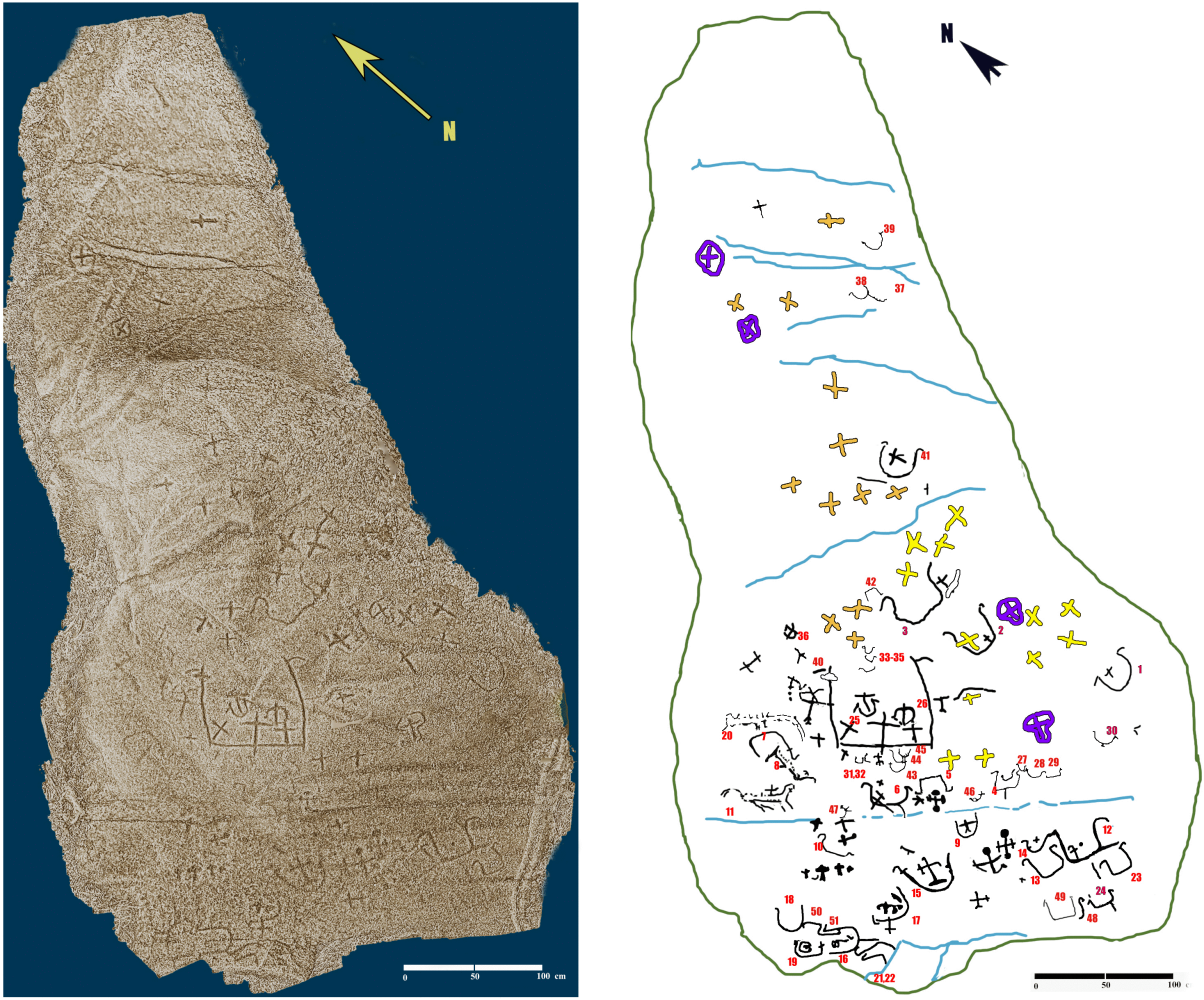
Photogrammetry (left) and line drawing (right) of the eastern panel at Borna. Reprinted from [181] under a CC BY 4.0 license, with permission from Antonio Costa, original copyright 2022. See also 3D model by ‘arqueoestela’: https://skfb.ly/6SOIX.

Both boats with a straight hull shape and more curved hulls shapes are represented (Figs 22,23), and it remains a possibility that some of the cruciforms inside are representations of masts [167]. Alonso Romero [179] described them as having a ‘swan neck’ and related this to animal heads at the end of Scandinavian boats. These boats have been compared with Scandinavian boats, alongside Egyptian, Mesopotamian, and the depictions at Mané Lud in Brittany [179] and consequently, have been variously interpreted as representations of hide boats [178–179], Aegean warships [169] and local plank-built boats [74].

The comparison with Scandinavian boat imagery [179] is well founded both with regards to the single line hull shape, whether slightly rounded or flat, the potentially high end-ships with their decorations of s-shapes or stylised animal heads (see, for example the two boats from Tanum 877:1 in Fig 3 and here: https://shfa.dh.gu.se/image/114489). Based on these features with a particular emphasis on the end ship decorations, the boat depictions at Borna might date to c. 1000–900 BCE (Figs 3,4).

### Bouça da Miséria

Monte de S. Romão (300 m elev.) is a hill situated on the right bank of the river Ave with good vantage views over the river [182]. The hill has steep slopes on its eastern and southern sides. Martins Sarmento first studied this site in the 19^th^ century. Since then, the site has been studied for decades, both by Mário Cardoso (in the first half of the 20th century) and by archaeologists from the Martins Sarmento Society and the University of Minho in the 21st century, particularly regarding its rock art [80–82,182–184].

**Fig 24:**
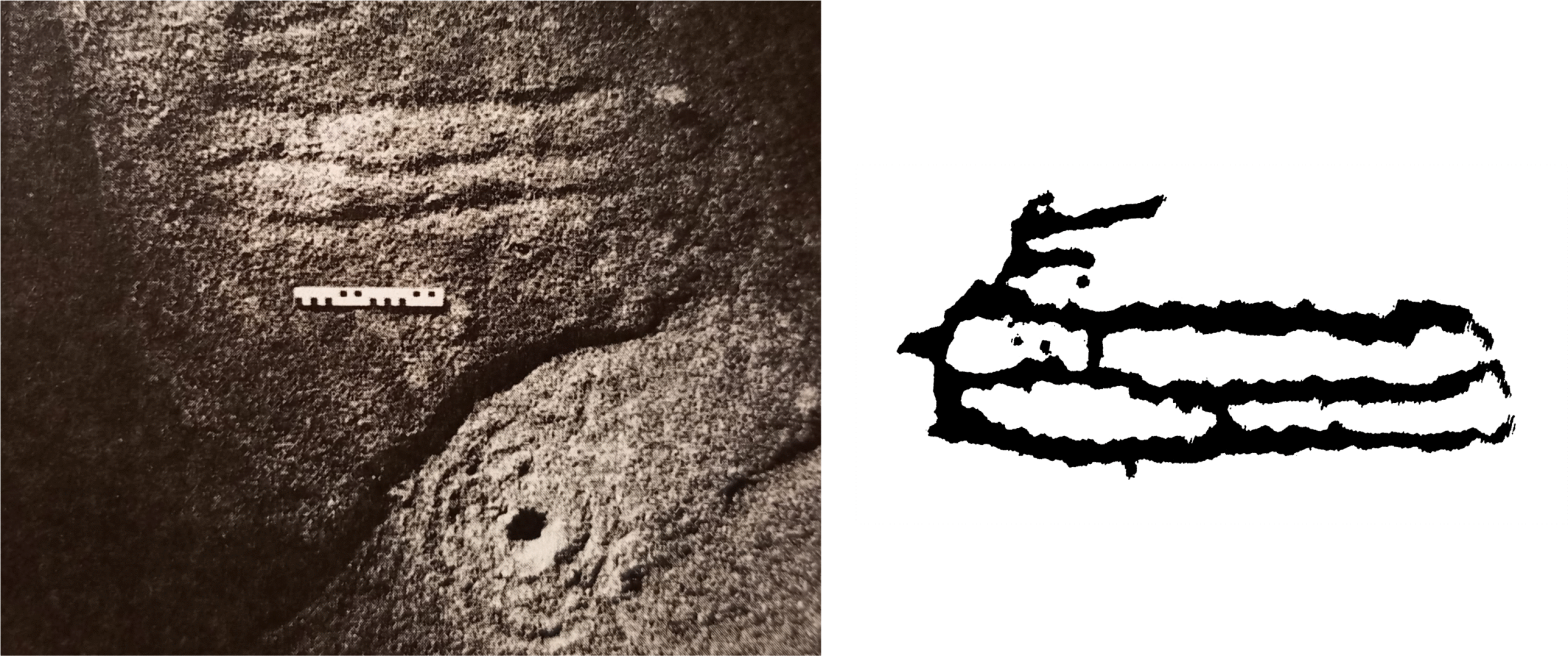
Boat of Bouça da Miséria (left) and details of carving (right). Reprinted from [80] under a CC BY 4.0 license, with permission from Daniela Cardoso, original copyright 2015.

There are currently 3 representations of boats coming from the hill, where the well-known Iron Age settlement of Briteiros sits, although Bouça da Miséria is outside the area of this archaeological site. The Bouça da Miséria outcrop is on an incline and is slightly eroded. The boat is found in the southwest corner made up of several lines (Fig 24) [80]. The almost brick-like shapes that makes up the hull of this vessel is reminiscent of some early BA Scandinavian boats (see the boat from Svenneby 19 in Fig 3), and might represent an early plank-built hull, despite the lack of prominent end-ships. The plank-built boat technology is first documented in Britain from around 2000 BCE and is generally associated with the trade in bronze metals. However, mobility related to the Bell Beaker phenomenon, the earliest evidence of which can be found in western Iberia, and metal‑exchange systems may have contributed to the conditions that enabled plank‑built boat innovation. Perhaps this boat image is one of the earliest depictions of this new technology?

**Fig 25:**
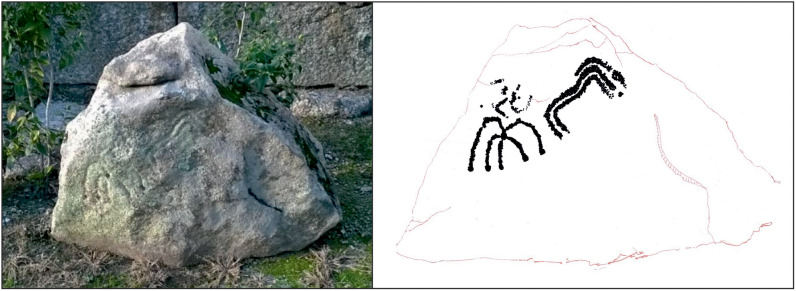
Engraved rock in the SMS Museum (left), next to line drawings of the engravings. Reprinted from [81] under a CC BY 4.0 license, with permission from Daniela Cardoso, original copyright 2020.

**Fig 26:**
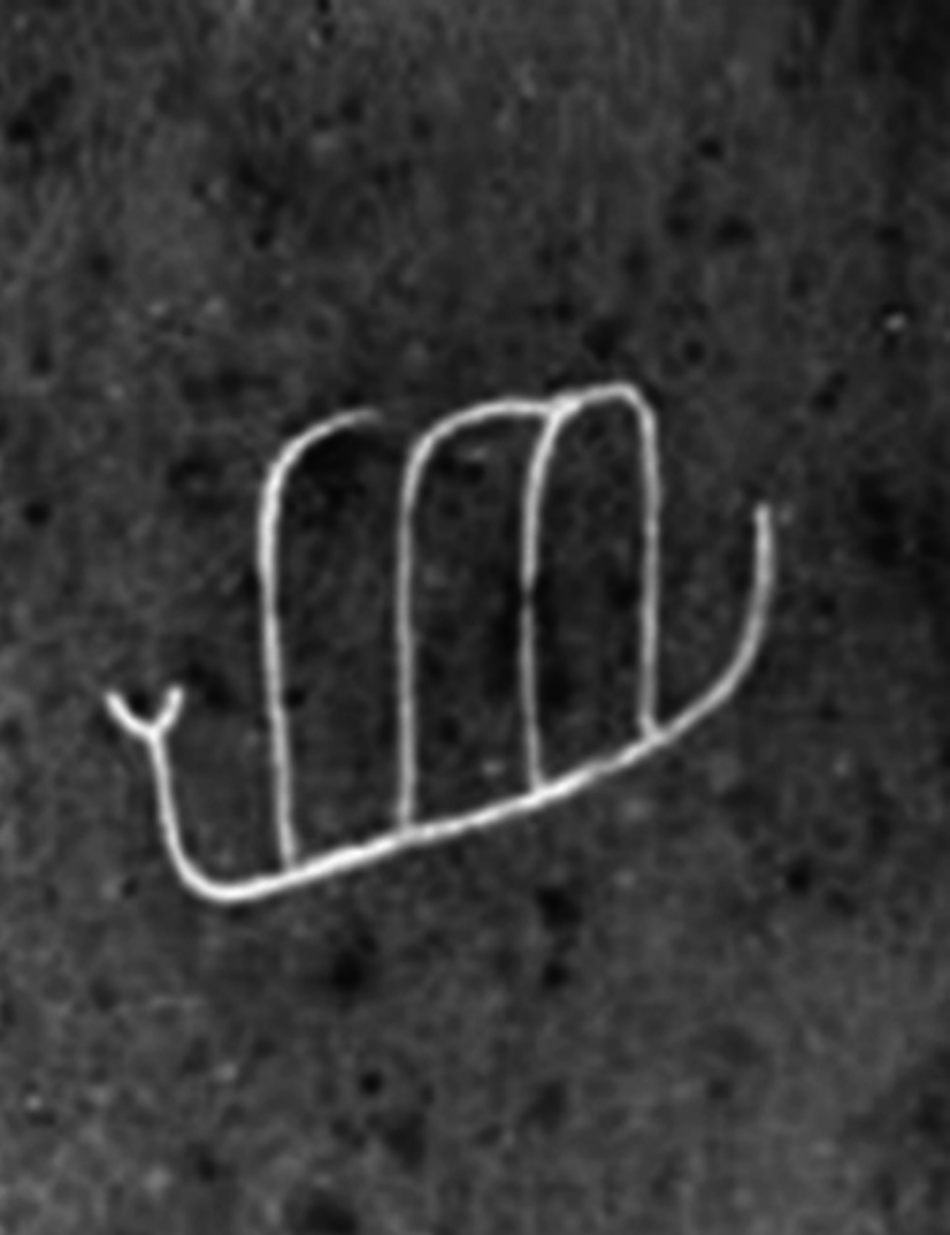
Sarmento’s boat 3 image. Reprinted from [81] under a CC BY 4.0 license, with permission from Daniela Cardoso, original copyright 2020.

Another engraving appears on a boulder found in the Archaeological Museum of the Martins Sarmento Society (Fig 25), that was originally part of a larger outcrop from the southern slope of Monte de S. Romão [185]. The third boat depiction (Fig 26) is only known through the recordings made by Martins Sarmento from the wall of a structured Iron Age settlement in Briteiros but has not been re-located [81].

Due to the high visibility of Monte de S. Romão, Cardoso [151] believes the boats were placed at a strategic point for navigating the river Ave for goods and trade. Cardoso [82,151] observes that the boat iconography is like representations in Scandinavia and Brittany which could link to long-distance interconnectivity and trade.

Whether the depictions found at the Monte de S. Romão site are meant to represent boats can of course be argued. In terms of the engravings on the boulder (Fig 25), the right-hand figure does suggest a boat shaped form, whereas the carving to its left might be interpreted as a fountain of life symbol, which has been noted on a Scandinavian razor dated to the LBA (see Fig 3: 10, with relevant Scandinavian parallels). A similar fountain of life symbol can be found on a rock art boat from Åmøy 3 in Norway.

As for boat 3 recorded at Monte de S. Romão (Fig 26), this is more likely to be the depiction of a boat. Similar rounded shapes as the three interlocking ones found amidships on this vessel can be found on several Scandinavian boats ranging from sites in Östra Eneby on the Swedish east coast to sites in Kville, Askum and Tossene on the Swedish west coast, but similar features can also be noticed on several boats on bronzes (Fig 3). One line of interpretation is that it is a representation of some sort of cargo being transported or tents erected to provide shelter for a sleeping crew [97]. Of interest here is also the v-shaped decorations at the top of the end-ships which, although rare, also appear in the Scandinavian rock art imagery (Figs 3, 26)

### Landscape setting: comparing Northwest Iberia with Scandinavia

**Fig 27:**
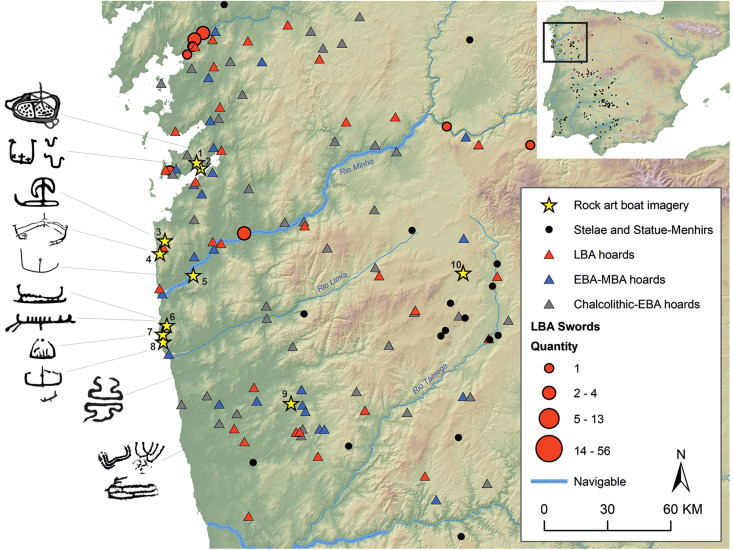
Distribution of rock art boat imagery in northwest Iberia: 1. Escada 2; 2. Borna; 3. Alto das Veigas; 4. Auga dos Cebros 1-3; 5. Senhora de Encarnação 1; 6. Santo Adrião; 7. Laje da Churra; 8. Eira do Louvado; 9. Bouça da Miséria, Monte de S. Romão / Boulder in Museum of Martins Sarmento Society; 10. Penedo do Muro 1 and 2. (Small black dots in small map also show the location of stelae and statue-menhirs). Reprinted under a CC BY 4.0 license, with permission from Marta Díaz-Guardamino. Shaded relief using the continental Europe digital terrain model from [148] (CC BY 4.0.).

Boat imagery is recorded in twelve rock art sites in northwest Iberia (Fig 27; Table 1). Perhaps unsurprisingly, most of the sites with boat imagery in NW Iberia are clustered directly on the Atlantic coast, in a small area spanning from the Ria de Vigo to the estuary of the river Lima, except for the Ave and Montes river valleys in the inland areas (Fig 27).

**Table 1:** Main landscape characteristics of open-air rock art sites with boat petroglyphs in northwest Iberia.

Name	County	Province	Country	Dist. coast (km)	Slope (deg.)	Panel	Orientation	Elev. (m)	Dist. to horizon (km)	Visibility to horizon
**Escada 2**	Monte Faro de Domaio	Vigo	ES	2.1	16	Flat but raised centre	SW	161	45	Faro Ravine, Moaña bay and the Atlantic Ocean.
**Borna**	Meira, Moaña	Pontevedra	ES	0.32	22	Flat but raised to the SW	S	102	36	Ria de Vigo and opposite coastline, El Latón bay, Cíes archipelago, and the Atlantic Ocean.
**Auga dos Cebros 1, 2, 3**	San Mamede de Pedornes	Pontevedra	ES	0.9	15	Flat	W	126	40	River Villar, Oia bay, and the Atlantic Ocean.
**Alto das Veigas 2**	Oia	Pontevedra	ES	2.9	7	Flat	NW	461	77	Visibility covers River Mougás and Laxes bay, and the Atlantic Ocean.
**Senhora de Encarnação 1**	Vila Nova de Cerveira	Viana do Castelo	P	15	14	Slight slope	SE	225	53	Minho River Estuary, Atlantic Ocean and Monte of Santa Tegra.
**Santo Adrião**	Caminha	Viana do Castelo	P	2.4	16	Vertical (side of large outcrops)	S	220	53	Âncora River Estuary, slopes of the Santa Luzia Mountain range, Afife beach, Âncora bay, and the Atlantic Ocean.
**Laje da Churra**	Viana do Castelo	Viana do Castelo	P	1.5	7	Slight slope	S-SE, S, S-SW, SW	67	29	Litoral plataform, Montedor promontory, slopes of the Santa Luzia mountain range and the Atlantic Ocean.
**Eira do Louvado**	Viana do Castelo	Viana do Castelo	P	1.2	8	Flat	W	33	20	Montedor promontory, slopes of the Santa Luzia mountain range, Areosa and Canto Marinho beaches, and the Atlantic Ocean.
**Penedo do Muro 1**	Monterrei	Ourense	ES	107	5	Flat	S	802	101	3 km visibility covers the Muiño and Barroca creeks and River Montes.
**Penedo do Muro 2**	Monterrei	Ourense	ES	108	7	Flat	S	800	101	3 km visibility covers the River Bocas, Muiño and Barroca creeks and river Montes.
**Bouça da Miséria**	Guimarães	Braga	P	40	7	Flat	SE	302	62	River Ave.
**Monte de S. Romão / Boulder in Museum of Martins Sarmento Society**	Guimarães	Braga	P	c. 40	(?)	(?)	(?)	(?)		The Monte de S. Romão has visibility to River Ave valley.

In fact, as GIS analysis indicates, many of the sites (7) are found within a 3 km radius of the Atlantic coast, with the closest only 0.32 km away as the crow flies (Petroglifo da Borna). There are also a few inland sites: Penedo do Muro 1 and 2, Bouça da Miséria, Monte de S. Romão boulder and slab of Monte de S. Romão. Both Bouça da Miséria and S. Romão are approximately 40 km from the coast, while Penedo do Muro is around 108 km away.

**Fig 28:**
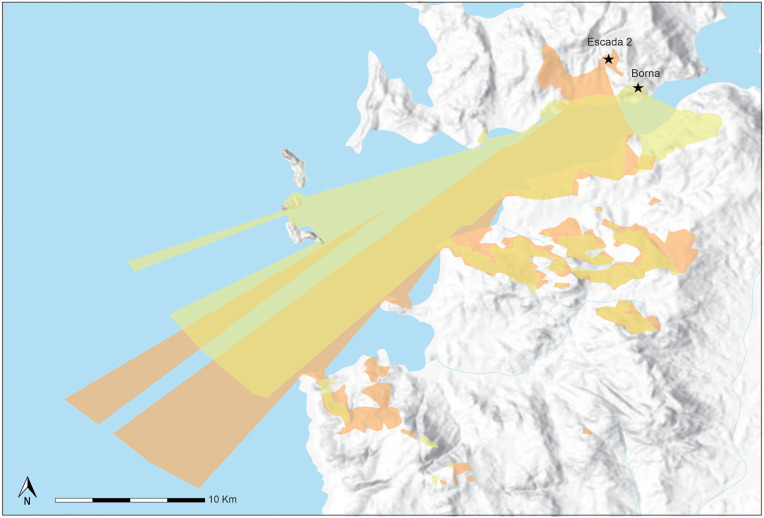
Distance to horizon viewsheds from the sites of Escada 2 and Borna, located north of the Ria de Vigo. Reprinted under a CC BY 4.0 license, with permission from Ellie Newton and Marta Díaz-Guardamino. Hillshade visualization using the continental Europe digital terrain model from [148] (CC BY 4.0.).

The combined GIS analysis of distance to horizon, viewshed and slope of individual sites (Table 1) reveals a systematic visual relation between rock art sites with boat imagery and bodies of water, primarily the sea but also rivers. The calculated distance to horizon of eight sites ranges between 36 km (Borna) and 101 km (Penedo do Muro), covering extensive sections of the surrounding coast, including key landing sites and river estuaries, and important portions of the Atlantic Ocean. Escada 2 and Borna, only 2.46 km apart, are an important case in point (Fig 28). Both sites have restricted views to the north and west. However, both are located on elevated positions (100 amsl), with panels orientated to the SW and S, with wide views over the Ria de Vigo and the nearby bays to the south. In fact, their viewsheds (up to their respective distance to horizon) fully visually control movement within the mouth of the Ria de Vigo and beyond (covering the Cíes archipelago too) (Fig 28).

**Fig 29:**
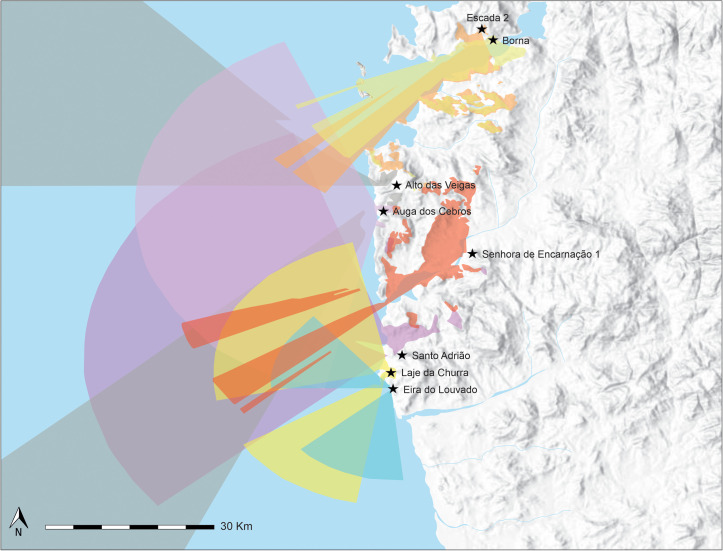
Distance to horizon viewsheds from the sites of Alto das Veigas 2, Auga dos Cebros, Senhora de Encarnação 1, Santo Adrião, Laje da Churra and Eira do Louvado. Reprinted under a CC BY 4.0 license, with permission from Ellie Newton and Marta Díaz-Guardamino. Hillshade visualization using the continental Europe digital terrain model from [148] (CC BY 4.0.).

**Fig 30:**
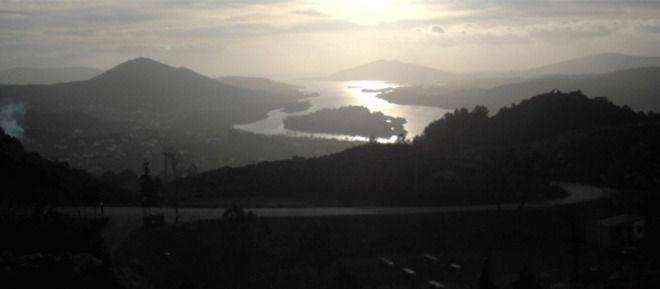
River Minho Estuary from Senhora da Encarnação 1. Reprinted under a CC BY 4.0 license, with permission from Ana M. S. Bettencourt.

Just off the coast of Oia, it is interesting to note the proximity between the sites of Auga dos Cebros and Alto das Veigas 2 (approx. 4.87 km) (Fig 29). The Alto das Veigas 2 panel is flat, and the site is located on high altitude (461 amsl) with commanding views to the northwest, focusing on the surrounding upland areas, and the coastline. From the site there is a visibility to horizon of 77 km, covering the River Mougás and Laxes bay, and the Atlantic Ocean. The Auga dos Cebros panels are on a slightly inclined slope orientated to the west, at relative low altitude (126 amsl), close to the coast, with commanding views to the west and southwest, covering the coastal front (Fig 29). Further inland (15 km) and south of the Minho River lies the site of Senhora da Encarnação 1, situated at a high elevation (225 m asl) and commanding extensive visual control over the Minho estuary (Fig 30).

**Fig 31:**
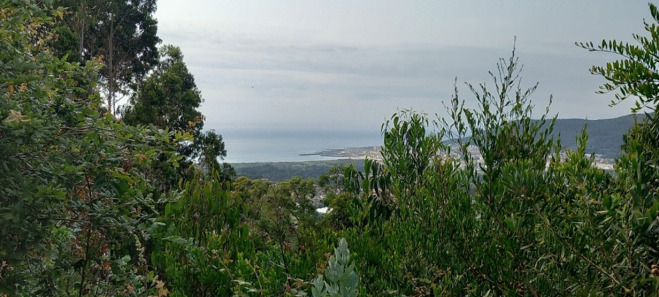
View of the Atlantic Ocean from the site of Santo Adrião. Reprinted under a CC BY 4.0 license, with permission from Hugo A. Sampaio.

**Fig 32:**
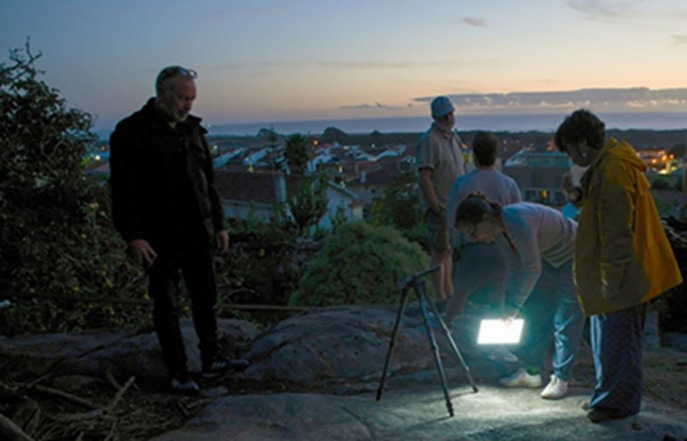
View of the Atlantic Ocean from the site of Eira do Louvado. Reprinted from Luís Coutinho under a CC BY 4.0 license.

North of the estuary of the river Lima, there is another cluster of sites with nautical iconography composed of Santo Adrião, Laje da Churra and Eira do Louvado, all located along the coast (Figs 29, 31–32). The sites are near one another with only 6 km between Santo Adrião and Eira do Louvado. The distance to horizon viewshed for these sites is quite extensive, with overlapping visibility to the west and north-west (Fig 29). Importantly, approximately 9 km of coast is visible between these 3 sites. Within this group of sites, the site of Santo Adrião stands out for its elevated position (220 amsl), with boat petroglyphs on the horizontal and vertical panels in the context of a set of large outcrops, with observer points that offer panoramic views to the northwest, west and southwest (Fig 31). Laje da Churra and Eira do Louvado, in contrast, are located closer to the coast at lower altitude, and while their boat petroglyphs are found on gentle slops or flat surfaces, in that order, the sites have commanding views over the coastline and extensive distance to horizon viewsheds (Table 1, Figs 29,32).

**Fig 33:**
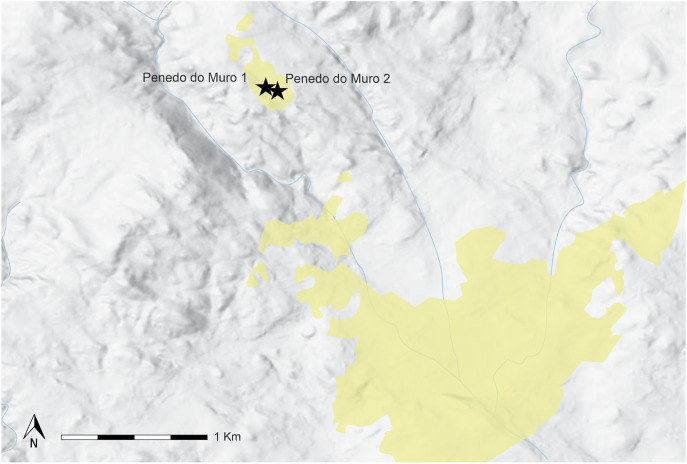
Cumulative viewshed from Penedo do Muro 1 and 2 (3 km). Reprinted under a CC BY 4.0 license, with permission from Ellie Newton and Marta Díaz-Guardamino. Hillshade visualization using the continental Europe digital terrain model from [148] (CC BY 4.0.).

Finally, the sites of Penedo do Muro 1 and 2 are located inland, at high altitude (c. 800 amsl), around 100 km away from the coast. The petroglyphs are found on flat panels, only 20 m apart. Curiously, there is limited visibility directly around the panels, while the shared visibility has considerable overlap and is focused on the southwest where the creeks Bocas o do Muíño and Carzoá de Barroca join the river Montes, which becomes the river Souto (Fig 33). Interestingly, the theoretical viewshed to the horizon coincides with the straight-line distance from the sites to the coast—approximately 100 km—which raises the question of whether the sea could be visible from the sites on exceptionally clear days.

These findings are not dissimilar to how the Scandinavian rock art is situated in the landscape. The southern Scandinavian rock art appears in clusters along the coasts and along rivers, where, in the latter case they are often found near important thoroughfares or rapids within river networks [186]. It has been estimated that over 70 percent of the southern Scandinavian rock art appears within 500 meters of the paleogeographic coast [187]. Within ‘cluster areas’, individual rock art panels are found to have been originally located within a range encompassing anything from right on the paleogeographic shoreline to more elevated locations, with occasional panels located several kilometres away from the coast. However, in almost all cases a connection between boat imagery and navigational waters is evident, whether within relatively sheltered archipelagos, along lee coasts (here defined as coasts that offer shelter in relation to the prevailing wind and wave direction), or rivers, and landing sites [96,98,110,188].

**Fig 34:**
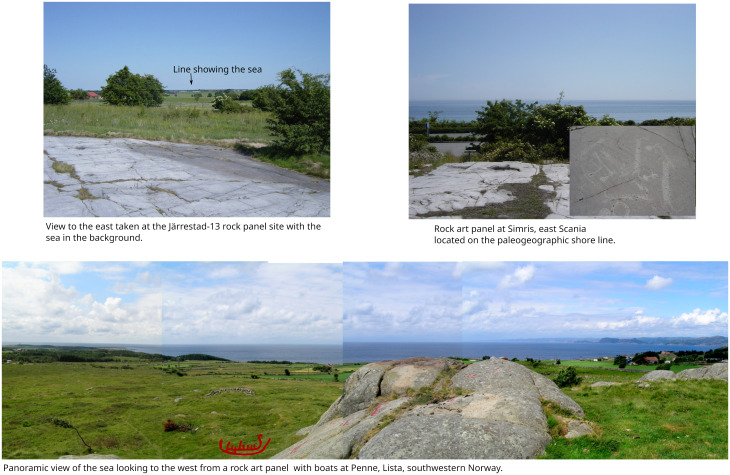
Examples of southern Scandinavian rock art sites with a clear connection to the sea and waterways. Reprinted under a CC BY 4.0 license, with permission from Boel Bengtsson. High quality digital documentation of rock art boat representations can be found at the Swedish Rock Art Research Archives here: https://shfa.dh.gu.se/.

**Fig 35:**
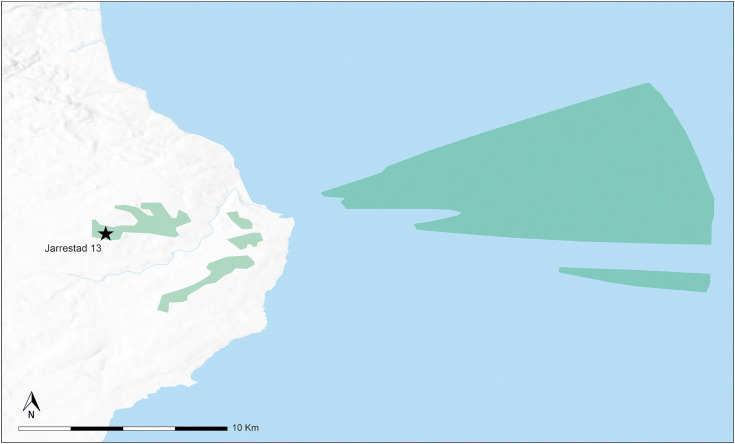
Viewshed to horizon from the site of Järrestad 13. Reprinted under a CC BY 4.0 license, with permission from Ellie Newton and Marta Díaz-Guardamino. Hillshade visualization using the continental Europe digital terrain model from [148] (CC BY 4.0.).

**Fig 36:**
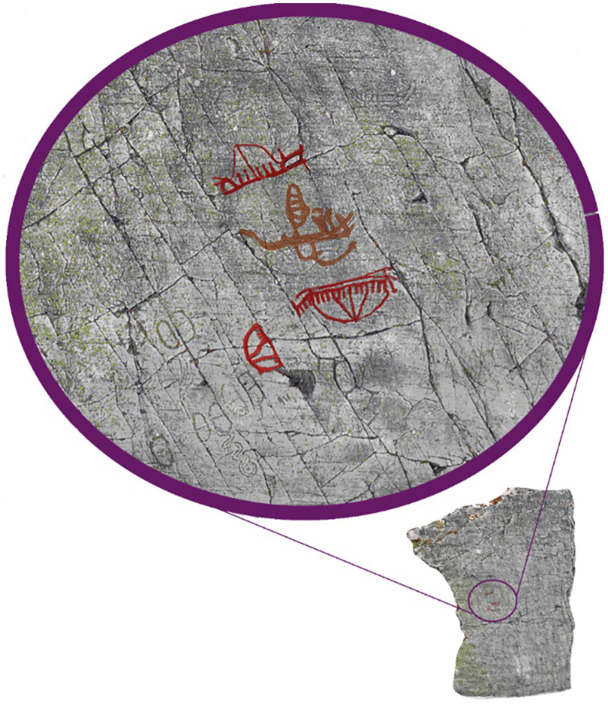
Bottom right, the 25 x 20 m large Järrestad 13 rock carving panel with imagery that date between 1700–200 BCE [94]. There are over 20 boats depicted on this panel but the three discussed in this article are highlighted in red, with a fourth boat with a sail like attribute highlighted in brown. Reprinted under a CC BY 4.0 license, with permission from Boel Bengtsson and Julián Moyano Di Carlo. High quality digital documentation of rock art boat representations can be found at the Swedish Rock Art Research Archives here: https://shfa.dh.gu.se/.

A prime example of boat imagery appearing on a panel located at a relative distance from the sea can be found at Järrestad in southern Sweden (Figs 34,35). The panel (Fig 34 and Fig 36) is located at a distance of c. 5 km from the paleogeographic shoreline to the east but on a clear day the sea is clearly visible from the site, offering an immediate connection to seafaring routes and landing sites [102]. The Tommarps river which flows past the panel c. 1.6 km to the south and continues its meandering path towards the sea and the Simris rock carving panel to the east (Figs 34,35), is believed to have been navigable up until the Viking Age [189]. This, as will be argued further in this paper might explain the presence here of some of the most extraordinary boat carvings in Scandinavia with clear parallels to Iberian imagery [102].

**Fig 37:**
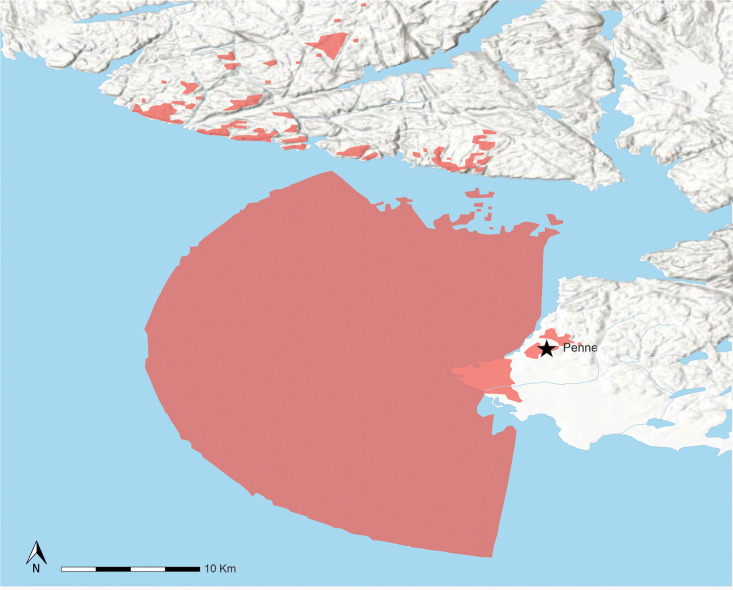
Viewshed to horizon from the site of Penne 13. Reprinted under a CC BY 4.0 license, with permission from Ellie Newton and Marta Díaz-Guardamino. Hillshade visualization using the continental Europe digital terrain model from [148] (CC BY 4.0.).

Another site that offers an even more dramatic view of what in the Bronze Age would have been important waterways can be found at Penne on the strategically located Lista Peninsula in SW Norway (Fig 37) [93]. This panel is located at an elevated position some 900 m from the paleogeographic shoreline and offers panoramic views to the west and to the west-northwest [93]. Thus, anyone standing here would have been able to see all southbound seafarers following the Norwegian coast coming down from the rich Bronze Age areas surrounding the Stavanger fjord almost 140 km away. This coastal route is comparatively exposed for seafaring, lacking a sheltering archipelago [190]. In combination with the relatively minimal sea level change in this area [191], the clear affinity between rock art boat depictions and natural landing sites along the coast and inland river systems becomes apparent. Although limited to only three sites, it might be significant that this rock art with boat imagery is found at two sites along the river Sokno (Kattaberget or Bø 56 and Haneberg 46), the mouth of which provides an excellent safe haven for boats, and at Egersund where they are carved onto a rock face facing away from the prevailing winds but within 300 m of the sea [104].

## Discussion

The recent digital recording, analysis and re-evaluation of boat petroglyphs from northwest Iberia, including their comparative analysis with Bronze Age boat imagery from Scandinavia, add valuable information about boat technology and capabilities in late prehistoric Iberia, the chronology of boat rock art depictions in the region, and their meaning and broader socio-economic and cultural context.

One of the main outcomes of the systematic formal comparison between southern Scandinavian boat iconography dated to the Bronze Age (1700−500 BCE) and western Iberian boat iconography presented in this paper is the identification of numerous parallels between the boat images found in the rock art of both regions. These similarities suggest that the Iberian depictions may be dated—at least approximately—to between 1200 and 500 BCE, corresponding to the Late Bronze Age and Early Iron Age in Iberia (Periods III to VI in Scandinavia). Older chronologies cannot be ruled out if we consider the sun cross that overlays a boat depicted with a mast at Laje da Churra, as sun crosses in Scandinavia may be dated from c. 1700−500 BCE (Periods I-VI) or later (e.g., sun cross on a Period III boat from Bottna (see [96: 76]) [21]. Also, parallels strongly suggest a shared iconography and imply direct or indirect cultural and technological exchange across vast distances. The Iberian boat depictions are very likely the first representations of Scandinavian boats identified outside Scandinavia, concentrated in a region that played a key role as a connectivity and exchange hub in Atlantic Europe during the Late Bronze Age.

Previous research has proposed various alternative hypotheses regarding the origin and chronology of Iberian rock art boat depictions, ranging from purely indigenous expressions to Mediterranean or even historical origins. Some authors have argued that these depictions represent autochthonous hide boats or extended logboats, supported by classical texts like Strabo describing the use of leather boats in northern Iberia before the arrival of the Romans [70,75,179]. Others have championed an eastern Mediterranean or Aegean origin, interpreting the vertical hull lines on boats like Auga dos Cebros as rowing benches typical of Late Helladic IIIB-IIIC Mycenaean or Late Cypriot IIC ships dating to 1325−1050 BC [165]. Similarly, early interpretations of vessels at sites like Borna suggested they were Egyptian-style reed bundle boats or Phoenician ships [70,169]. Finally, a few scholars have even suggested medieval or modern historical chronologies for Borna [192: footnote 1], arguing that the complex composition, deep V-shaped grooves, or deviation from the typical regional rock art tradition indicate recent execution, such as parish boundary markers or historical graffiti. However, a recent photogrammetric study [181] refutes this by confirming that the grooves were not made with sharp metal tools. Many show no erosion because the rock surface remained buried and protected until the site was uncovered. Since the discovery, however, people have re‑carved some of the grooves with hard stones to make them more visible, a damaging practice that has increasingly deepened them. Finally, the site’s use as a parish boundary marker does not justify assigning a historical date to the entire complex. While the crosses may be later additions, most carvings on the panel can still be attributed to a prehistoric phase.

Overall, the application of high-resolution 3D documentation and systematic comparative analysis provides strong evidence to refute these alternatives in favour of Late Bronze Age Scandinavian connections. While indigenous communities undoubtedly possessed a rich nautical heritage prior to Phoenician contact, the complex rigging, masts, arched stays, and highly elevated end-ships adorned with animal heads or S-shapes are structurally incompatible with simple hide or reed vessels. Furthermore, while Mediterranean contacts certainly existed, the specific typological details revealed by the photogrammetry—such as the “keel-to-keel” layouts and the recurrent association with segmented circles (sun crosses) and equids—are intrinsic hallmarks of Nordic Bronze Age solar mythology and iconographic traditions. Exact iconographic matches, such as the *Järrestad 13* panel in Sweden which shares the same mast configurations and arched stays as the Iberian examples, firmly root these depictions within a shared Late Bronze Age Atlantic-Scandinavian network rather than an exclusively Aegean, Egyptian, or historical context. This provides significant new insights, as previously, Bronze Age boats depicted on rocks were commonly and predominantly associated with Scandinavia, where over 20,000 boat depictions have been discovered.

The perhaps strongest indication of potential direct contact between these two regions is provided by the new interpretation of boat carving from Santo Adrião as of Scandinavian design dated to period III (c. 1300−1100 BCE). This, however, is not the only boat depiction present within the material presented here that convey striking iconographic similarities between the two regions. At Penedo do Muro, e.g., we find the outlines of several boats depicted in a ’keel-line to keel-line’ position in the same way as frequently appearing on Scandinavian bronze artefacts such as razors and neck rings from period VI (700−500 BCE). At Alto das Veigas 2 it is the boat with a ’mushroom’ amidships that suggest an affinity to Scandinavia where the equivalent symbol can be dated from c. 900−500 BCE. Whereas in the Scandinavian material such symbols amidships are usually associated with boats travelling to the right, there is at least one exception which offers a direct parallel to the Alto das Veigas boat. Alternative interpretations of this symbol include the possibility it represents a ceremonial axe or a sail. Perhaps it is even a combination of an axe and sail that is being referred to; the use of a boat and a sail to acquire the treasured ceremonial axe? In this scenario the sun, which frequently accompanies this symbol on the bronzes and appear inside boats within the rock art (Figs 7,10,19) might symbolise its importance as a navigational aid in long-distance seafaring ventures [92,146]. The presence of these sun crosses also in the Iberian rock art imagery, and where the symbol appears near boats could also perhaps refer to the significance of the sun for navigation.

Also, at Auga dos Cebros we find a boat depiction featuring several details that demonstrate an affinity with Scandinavian boat imagery. These details include the use of vertical hull lines, a prow decorated with a likely horse’s head and a stern decoration of an inwards facing curl typical of Scandinavian boat imagery of Period IV (1100−900 BCE). In addition, the mast and way in which the stays on this boat are conveyed as slightly arched lines as if to suggest they are non-rigid, is a trait shared with the Scandinavian imagery. Whereas horse’s heads first appear within the Scandinavian iconography in period II (1400−1300 BCE), all these traits together would suggest a comparable date of the Auga dos Cebros boat of somewhere around 1200/1100 BCE.

Parallels with Scandinavia show that the technology and capabilities of boats depicted in NW Iberia could be advanced and quite varied. There are boats depicted with oars and crews comparable to those in Scandinavia (Santo Adrião, Eira do Louvado, Laje da Churra), and masts and vertical or s-shaped end-ships like those in Nordic rock art (Laje da Churra, Eira do Louvado, Penedo do Muro 2) (Fig 3). Some boats with s-shaped end-ships that can also be found within the Nordic rock art show Nordic-style boats are also found in more than one site (Laje da Churra, Escada 2, Borna). Auga dos Cebros and Laje da Churra include the clear depiction of a bow and curved stern, steering oars, mast and rigging, sail, curved hulls, and plank boats, traits that can also be found in Scandinavia.

When comparing the Scandinavian and the west Iberian iconographic material it becomes evident that sail as a method of propulsion is likely to have been widely used along the Atlantic European façade in the Bronze Age. Whereas the earliest Scandinavian boat imagery, a single masted vessel from Vendsyssel, Denmark, dated to c. 1550 BCE (Fig 2), boat 1 from Auga dos Cebros is likely to date from around 1200 BCE (Fig 13). This is not very different from the c. 1300−1100 BCE date suggested for Auga dos Cebros 2 (Fig 24), which, although showing potential affinity with Mediterranean boat iconography also has a mast supported by stays [169]. Other boats that imply the use of sail in the region in the Bronze Age can be found at Eira do Louvado and Laje da Churra. At the latter site in particular one more round hulled boat on panel 6 stands out, featuring what could be interpreted as mast with a yard, supported by stays. The hull shape of this boat is like Agua dos Cebros 1 which could indicate a similar date. In comparison, the Lisbon pottery sherd with its decoration of a potentially Phoenician boat [158] is furnished with an unmistakable mast which appears to be taller. Except for the three boats from Järrestad and a two-masted boat on a now lost bronze buckle, this is the most common way of depicting masts within the Scandinavian iconography. The mast lengths on most of the sailed vessels both in the Scandinavian and some but not all the Iberian iconographies, suggest relatively low aspect ratio rigs were used. This is also the case on a peculiar boat from Bottna, west Sweden, which bears some resemblance to the Lisbon boat but has a shorter mast.

At Laje da Churra there are also a multitude of vessels that appear to be rowed. Since this method of propulsion generally require wider boats to be used efficiently, such depictions are unlikely to represent logboats. Other technical details found on this panel are the many different positions of steering oars, something that is also the case within the Scandinavian boat imagery and likely reflect the hands-on practicalities of keeping directional control of a boat.

It is now clear that most of the Scandinavian rock art boats are likely to represent plank-built vessels with the oldest such boats dated already in period I [146]. The plank-built boat technology is first documented in Britain from around 2000 BCE and is commonly associated with the emergence of bronze metal trade [193–194]. However, both the north Atlantic trade networks and the spread of the Bell Beaker phenomenon—first identified in western Iberia in the first half of the 3rd millennium BCE [158,195,196]—may have been connected to the earlier emergence of plank‑built boat technology. The other known boat-building tradition used along the north Atlantic façade, at least as far back as the 6th century BCE but believed to have roots already in the Neolithic, is the hide boat built on basket-weave technology [158–160,194].

By comparison, the potentially earliest boat representation within the western Iberian boat iconography investigated here might be one of the boats from Bouça da Miséria. Its brick like appearance is reminiscent of early Bronze Age boats in Scandinavia and might reflect an early form of plank-built boat technology. This study also provides an opportunity to reflect on some of the simpler Scandinavian boat imagery that is rarely discussed, perhaps because of the abundance of more elaborate boat depictions. In view of the Iberian rock art where such depictions are relatively common, it is clear this imagery has much more to offer for the understanding of boats in prehistory. As we can see in Fig 3 this imagery includes not only different positionings of steering oars but also many other curious details that might related to, e.g., cargoes.

The disparity in boat depictions along the northwestern coast of Iberia may stem from their being engraved at different times, by various individuals, and influenced by distinct regions of Scandinavia. Alto das Veigas 2 and Penedo do Muro 2, for instance, exhibit notable similarities to imagery from the Danish region. It is also worth considering the hypothesis that certain panels—such as those at Santo Adrião or parts of Laje da Churra—represent scenes featuring multiple types of boats, of either foreign or local origin, as previously suggested by Bettencourt [14–15] and Santos-Estevez & Bettencourt [2]. All vessels featuring masts, rigging, and flattened hulls are likely of foreign origin and intended for long-distance voyages. Nonetheless, even the smaller flat-hulled boats—propelled by oars and crewed by sailors—typically exhibit prominent bows and sterns, a characteristic well-suited to Atlantic navigation [2,14,15].

The ship depictions in the rock art of northwestern Iberia may have been created by local communities engaged in long-distance trade and exchange, reflecting their perceptions—or those of foreign or Iberian travellers—within a context of ‘early globalization’ marked by increasing supra-regional contacts [5,14,83,197]. If indigenous communities carved the precise typological details seen in the boat depictions of northwest Iberia, this may indicate that they were not merely observing foreign ships from afar but were fully integrated into these trans-regional maritime networks. They may have possessed an intimate understanding of naval architecture because they were expert navigators themselves, likely adopting, crewing, or building these vessels through technological transfer. Indigenous communities already maintained a millennia-old nautical tradition and a genuine hybridization of naval techniques, in which local shipwrights incorporated foreign innovations into their own boat-building practices [70,75,84].

Alternatively, these engravings might materialize cultic expressions of foreign origin, produced by travellers acting within their own ideological frameworks, yet interacting with local populations. The unusually detailed representation of the boat at Auga dos Cebros 1 (unlike the more typical local deer motifs) has led scholars to suggest that it was the work of a small group of foreign sailors or merchants, possibly carved as an *ex-voto* in gratitude for a safe landing or survival of a shipwreck [73]. This interpretation is questioned by Mederos [165], who notes that Oia is not a favourable landing site. However, many of the other boat depictions from northwest Iberia are located close to the coast, where numerous suitable landing sites exist. Furthermore, their presence at regionally significant ceremonial sites that incorporate indigenous iconographies suggests possible syncretic phenomena [14]. Outsiders or travellers arriving by sea might have chosen locally significant, previously engraved sacred sites to express their own beliefs (such as solar journeys). This could apply to sites including Auga dos Cebros 1, Escada 2, Santo Adrião, Laje da Churra, and Penedo do Muro 1 and 2. Other boat depictions appear in longstanding ceremonial contexts (e.g., Bouça da Miséria and Alto das Veigas 2), while some occur at *ex nihilo* locations lacking previous iconographic traditions (e.g., Auga dos Cebros 2 and 3, Borna, and possibly Eira do Louvado). Supporting the involvement of non-local agents is the discovery of rolled pebbles and other lithic artefacts lodged in the crevices of outcrops at Santo Adrião [2]—a practice also documented in the Bohuslän region of Sweden, where lithic and ceramic materials have similarly been found in Iron Age engraving sites [98].

What did these boats represent? They were not only means of transport but also carried mythological significance [5,14,83,198]. In some cases, they may have functioned as ideograms that express the concept of ‘journey’—both in its literal sense and as a metaphor for experiences shared by communities, including those related to the cosmos, such as mythical narratives tied to the sun’s path across the sky and journeys among the stars [14].

Interpreting these narratives in northwestern Iberia requires examining the spatial context of motifs on engraved surfaces—their orientation and associations—a process already underway in Portugal [2,14,21,76]. At Laje da Churra, for instance, the orientation of the boats is highly significant: they span from southeast to southwest panels, a layout suggestive of the solar journey, likely corresponding to summer [2]. This interpretation is reinforced by three segmented circles linked to the largest southeast boat—alongside a quadruped associated with one of these circles [21]—as well as two quadrupeds depicted with boats in the southwest panel [77]. The iconography of a solar boat is further supported by a cup mark on the largest southwest boat at Laje da Churra, a comet-like motif to the northwest of the principal boat at Santo Adrião, and cup marks positioned on the mast and to the northwest of the boat at Eira do Louvado. These elements appear to share parallels with Nordic iconography [2,14,21]. Solar iconography is also perceptible at Alto das Veigas 2, where cup marks and a comet-like motif surround the boat. A similar interpretation may apply to Auga dos Cebros 1, where quadrupeds encircle the boat, primarily in the northeast and southeast directions.

**Fig 38:**
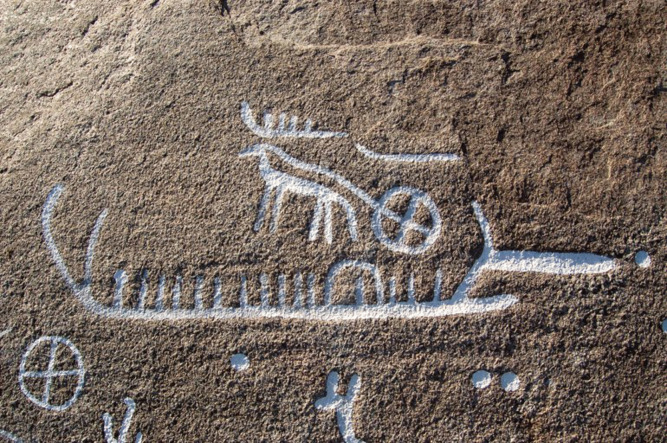
Kalleby 418 (#underslösmusem). Reprinted from Gerhard Milstreu under a CC BY 4.0 license.

The association between boats and solar mythology is paralleled in the Scandinavian Bronze Age [14,198]. Scandinavian research has long interpreted certain boat motifs as representations of the sun [160]. In recent years, Flemming Kaul has expanded this idea, arguing that Bohuslän rock art depicts actual ritual events related to the sun, performed in the Bronze Age landscape [92,198]. Kaul notes visual similarities between ship images on rock surfaces and those on bronze objects and in graves, but he distinguishes their meanings: rock art scenes reflect lived rituals, whereas bronze and grave depictions are more cosmological and normative [199]. Nevertheless, Kaul emphasizes the interaction between these spheres, suggesting that ritual actions were reinforced by celestial symbols such as sun crosses, wheels, horses, cup marks, and concentric circles. Bohuslän, in particular, contains numerous depictions interpretable as solar symbols—for example, at Bro 703, Bottna 334, Tanum 105:3, and Tanum 255. Fig 38 shows a sun horse above a boat, strongly reminiscent of the famous Trundholm sun chariot, dated to about 1500–1300 BCE [92]. The representation of ‘keel to keel’ position of boats, found in Penedo do Muro 2, can also be found in Scandinavia not only in rock art but also on bronze artefacts, where they are seen as elements of key cosmological value.

The varied configurations of ship images in Scandinavian rock art may reflect a broader shift toward maritime life during the Bronze Age. The sea likely drew diverse groups due to its demanding seasonal labour as well as the freedom and livelihood it offered. Some individuals may have alternated seasonally between land-based and maritime roles, while others maintained more permanent positions. Rock art may have served to mark or commemorate these transitions in social or occupational identity. Coastal rock art sites likely functioned as strategic points within regional seafaring networks—places for rest, resupply, and ritual. Their placement and symbolic imagery suggest they were settings for seasonal gatherings, initiation rites, and elite pilgrimages tied to broader transregional seascapes [99]. The numerous engraved ships may also reflect the ritual activities of secret societies that played a key role in maritime trade and elite social structures. Composed of warriors, travellers, and boat builders, these groups controlled surplus and exchange through esoteric knowledge, ritual paraphernalia, and symbolic practices [110]. Iconographic parallels between ship-shaped bronze razors and carvings of ships, warriors, and ritual attire—such as bird masks and horned helmets—support the view that rock art sites were used in initiation rituals linked to adulthood, warriorhood, and maritime institutions. Notably, warrior and ship motifs cluster in Bronze Age Periods II and V, coinciding with peaks in long-distance metal trade and suggesting institutional links between exchange, initiation, and rock art production [110].

In NW Iberia, the rock art sites discussed in this paper may be classified as ‘contact’ rock art, given their strategic locations—often isolated from domestic settlements but positioned near the sea, other bodies of water, and tin resources. The latter has been tested through GIS-based spatial analysis for the sun crosses (also known as segmented circles), a symbol of Scandinavian origin. Notably, 38% of the engraved outcrops with these types of motifs in the NW of Portugal lie within a theoretical two-hour walking distance of tin deposits [21]. Systematic GIS analysis of rock outcrops bearing maritime iconography reinforces their connection to maritime trade routes. Their proximity to the seashore, coupled with expansive horizon viewsheds, highlights the strategic significance of the Rías Baixas and the northern Portuguese coastline as key nodes of connectivity. These areas served as crucial landing sites and hubs for commercial and cultural exchange between distant communities, including those of Iberia and Scandinavia.

These regions facilitated interactions between geographically distant communities during the Late Bronze Age and Early Iron Age—particularly during Periods IV and V in Scandinavia [4]. This is evidenced by the widespread circulation of copper (and likely tin) originating in Iberia during the Late Bronze Age. From southern Iberian mining districts—such as Linares, Los Pedroches, and the Alcudia Valley—copper reached northwestern Iberia (e.g., ingots in northern Portugal, palstaves in Galicia; [66–67]), southern England (e.g., Salcombe Bay ingots; [68]), and Scandinavia [4,6]. The flow of copper followed extensive terrestrial networks—some spanning more than 800 km—traceable through the distribution of iconography and material culture within Iberia, such as warrior stelae and bronze axes [4,69].

In this context, rock art reflects not only the physical world and transportation technologies, or certain maritime institutions that made the sea journeys but also the symbolic and spiritual relationships between distant communities and their shared understanding of the sea and travel. But what about Penedo do Muro? Situated around 800 meters above sea level and approximately 100 km from the coast, its location appears anomalous. Penedo do Muro 1 and 2 lie in the Tâmega Valley, a region rich in tin resources [3]. These sites may signal the presence of visitors of Scandinavian origin, perhaps drawn by the search for tin or the desire to establish exchange agreements. In gratitude for a successful voyage, and through interactions with the indigenous population, these travellers may have chosen a local sanctuary—imbued with native iconography—as the place to inscribe their worldview. As northwestern Iberia emerged as a meeting point for copper trade between the southern regions and the broader Atlantic world, it was natural for communities to recognize and capitalize on local resources—such as tin and salt—which were actively exploited along this coast [200–202]. Rock art featuring ship imagery across Scandinavia, as well as along the coastal strip of Portugal and northwestern Iberia, reflects a broader Bronze Age shift toward maritime life. These carvings likely marked social or occupational transitions tied to seasonal seafaring, trade, and ritual activity. Their placement in strategic coastal locations suggests they served as sites for initiation, elite gatherings, and symbolic expression linked to maritime institutions and esoteric knowledge for groups that were engaged in long distance exchange for metals. The shared iconography across regions points to parallel practices and beliefs among seafaring communities along Europe’s Atlantic and Nordic coasts.

Ultimately, the exact typological parallels observed in the rock art, such as identical Nordic-style end-ships or Mediterranean rigging, demand a level of intimate knowledge that contradicts mere superficial ‘perception’ by isolated locals. Whether these depictions were carved as *ex-votos* by foreign crews landing on Iberian shores [14,73], or by indigenous seafarers who had fully assimilated foreign naval technologies into a hybridized maritime tradition [70,75,84], both scenarios confirm the same historical reality: the coastal communities of Northwest Iberia were deeply and directly engaged in intensive, long-distance maritime networks.

## Conclusion

This research prompts a re-evaluation of the understanding of rock art boat depictions in northwest Iberia, positioning them as integral elements of Atlantic long-distance connections likely dating to the Late Bronze Age. By leveraging advanced digital documentation (including 3D modelling, RTI and GIS) and systematic comparative typology with the established Scandinavian Bronze Age corpus, this study offers strong iconographic parallels consistent with extensive transregional exchange and maritime connectivity.

The core iconographic finding is the identification of numerous and striking typological and iconographic parallels between the imagery in northwest Iberia and Southern Scandinavia. Based on these similarities, we suggest a plausible chronological range for the Iberian examples of circa 1200–500 BCE, corresponding largely to the Late Bronze Age in Iberia (Scandinavian Periods III to VI). This shared iconography implies the existence of a robust, supra-regional interaction network capable of facilitating the movement of commodities, technological innovations, and complex ideological frameworks. The high technical detail revealed by the documentation provides new insights into Bronze Age maritime capabilities, confirming the depiction of advanced vessels. The imagery includes features suggesting the use of oars, crew, masts, rigging (often depicted with arched stays), and curved hulls, strongly supporting the hypothesis that sail technology was widespread along the Atlantic European façade during this period. Moreover, the presence of shared cosmological elements, such as sun crosses (segmented circles) near boats, suggests parallels with Nordic iconography and a shared preoccupation with solar mythology and the concept of ‘journey’.

The GIS-based landscape analysis substantiates the crucial link between the iconography and navigable waters. The sites, whether coastal or far inland, maintain a systematic visual or physical relationship with the Atlantic Ocean or major river systems. Coastal clusters possess expansive horizon viewsheds that visually control movement across key landing sites and estuaries (such as the Ria de Vigo and Minho River Estuary), reinforcing their strategic significance as hubs for commercial and cultural exchange. This pattern of placement—near the coast or major river thoroughfares—is highly comparable to nautical rock art clusters found in Southern Scandinavia. Even inland sites like Penedo do Muro, situated in a tin-rich valley and featuring Scandinavian-style iconography (e.g., ‘keel-to-keel’ positions), suggest that foreign agents, potentially seeking tin, interacted deeply with local populations.

Collectively, these findings directly challenge the dominant “terrestrial narrative” of European prehistory, establishing Atlantic Europe as a cohesive, navigable seascape during the Late Bronze Age. The rock art itself is interpreted as material evidence for “maritime institutionalization,” where long-distance exchange was managed and reflected by specific social groups (e.g., warriors, travellers). The concentration of these images confirms northwest Iberia’s centrality as an active maritime nexus connecting Mediterranean and Atlantic trade routes, particularly during periods of intensified metal trade (LBA Periods IV and V). While acknowledging the inherent limitation of relying on stylistic and comparative typological dating for the Iberian material, future research should focus on utilizing Lead Isotope Analysis (LIA) of Late Bronze Age metalwork to empirically ground the movement of metals along the routes suggested by the iconography. Furthermore, integrating detailed paleogeographic models of coastline changes will be essential to accurately map Bronze Age landing sites and navigable river sections.

In short, the enduring images carved into the rocks of northwest Iberia and Scandinavia inscribe the convergence of advanced seafaring technology, crucial economic connectivity, and shared cosmological worldviews across thousands of kilometres, offering a definitive testament to northwest Iberia’s pivotal role in Europe’s early global trade networks.
